# Identification of an algal xylan synthase indicates that there is functional orthology between algal and plant cell wall biosynthesis

**DOI:** 10.1111/nph.15050

**Published:** 2018-02-20

**Authors:** Jacob Krüger Jensen, Marta Busse‐Wicher, Christian Peter Poulsen, Jonatan Ulrik Fangel, Peter James Smith, Jeong‐Yeh Yang, Maria‐Jesus Peña, Malene Hessellund Dinesen, Helle Juel Martens, Michael Melkonian, Gane Ka‐Shu Wong, Kelley W. Moremen, Curtis Gene Wilkerson, Henrik Vibe Scheller, Paul Dupree, Peter Ulvskov, Breeanna Rae Urbanowicz, Jesper Harholt

**Affiliations:** ^1^ Department of Plant Biology Michigan State University East Lansing MI 48823 USA; ^2^ DOE Great Lakes Bioenergy Research Center Michigan State University East Lansing MI 48823 USA; ^3^ Department of Biochemistry University of Cambridge Cambridge CB2 1QW UK; ^4^ Carlsberg Research Laboratory 1799 Copenhagen V Denmark; ^5^ Complex Carbohydrate Research Center University of Georgia 315 Riverbend Road Athens GA 30602 USA; ^6^ BioEnergy Science Center Oak Ridge National Lab Laboratory Oak Ridge TN 37831 USA; ^7^ Department of Plant and Environmental Sciences University of Copenhagen 1971 Frederiksberg C Denmark; ^8^ Botanical Institute Department of Biological Sciences Universität zu Köln Köln D‐50674 Germany; ^9^ BGI‐Shenzhen Beishan Industrial Zone Yantian District Shenzhen 518083 China; ^10^ Department of Biochemistry and Molecular Biology Michigan State University East Lansing MI 48824 USA; ^11^ Joint BioEnergy Institute Emeryville CA 94608 USA; ^12^ Environmental Genomics and Systems Biology Division Lawrence Berkeley National Laboratory Berkeley CA 94720 USA

**Keywords:** biosynthesis, cell wall, evolution, IRX10, *Klebsormidium flaccidum*, *Klebsormidium nitens*, xylan, XYS1

## Abstract

- Insights into the evolution of plant cell walls have important implications for comprehending these diverse and abundant biological structures. In order to understand the evolving structure–function relationships of the plant cell wall, it is imperative to trace the origin of its different components.
- The present study is focused on plant 1,4‐β‐xylan, tracing its evolutionary origin by genome and transcriptome mining followed by phylogenetic analysis, utilizing a large selection of plants and algae. It substantiates the findings by heterologous expression and biochemical characterization of a charophyte alga xylan synthase.
- Of the 12 known gene classes involved in 1,4‐β‐xylan formation, XYS1/IRX10 in plants, IRX7, IRX8, IRX9, IRX14 and GUX occurred for the first time in charophyte algae. An XYS1/IRX10 ortholog from *Klebsormidium flaccidum*, designated *K. flaccidum* XYLAN SYNTHASE‐1 (*Kf* XYS1), possesses 1,4‐β‐xylan synthase activity, and 1,4‐β‐xylan occurs in the *K. flaccidum* cell wall.
- These data suggest that plant 1,4‐β‐xylan originated in charophytes and shed light on the origin of one of the key cell wall innovations to occur in charophyte algae, facilitating terrestrialization and emergence of polysaccharide‐based plant cell walls.

## Introduction

A large diversity of cell wall structural variations exist within the plant kingdom (Domozych *et al*., 2007; Harholt *et al*., 2012). One important avenue for understanding this diversity is by studying wall evolution and the rise of new architectural solutions and principles. Intriguingly, all plant cell walls are largely based on the same limited number of polysaccharide classes. At the same time, it is clear that some cell wall functions can be fulfilled by different architectural solutions, for example involving different groups of polysaccharides (Carpita & Gibeaut, 1993; Harholt *et al*., 2012). This suggests that simple carbohydrate composition gives a limited perspective of wall structure, whereas the combinatorial aspects of polymer–polymer interactions are the key players that define architectural complexity. How and why these different interactions evolved, including their specific relationships to wall architecture and function, remain enigmatic. However, before we can answer these questions it is necessary to establish the key routes of cell wall evolution by determining the evolutionary origins of the different cell wall polysaccharide classes to fill in current gaps in our knowledge.

**Figure 1:**
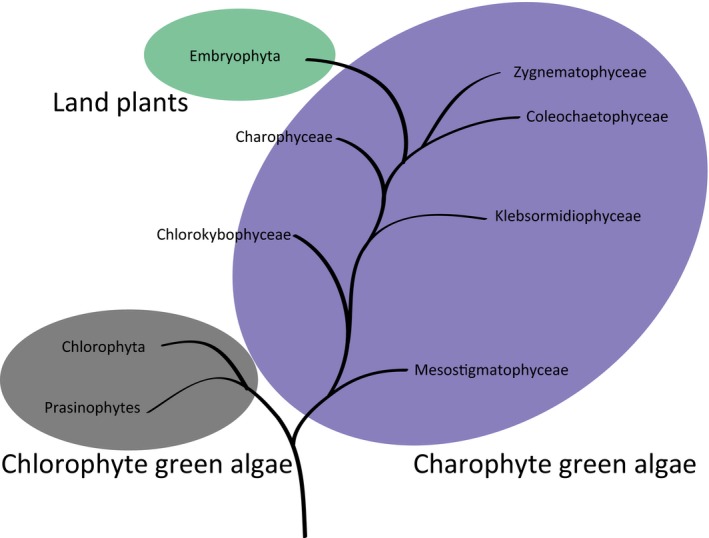
Phylogenetic relationship within Viridiplantae as suggested by Wickett *et al*. (2014). This clade is defined by chloroplast descendence, the name translating as ‘green plants’. It designates green algae and land plants. The algal species *Klebsormidium flaccidum* is a member of Klebsormidiophycea, located centrally in the grade of charophyte algae, while Zygnematophyceae is located as the most recently evolved group in the charophyte algae grade.

A direct ancestor of land plants (embryophytes) has not been identified, but the Zygnematophyceae (Fig. 1) have been proposed as the closest extant relatives (Wickett *et al*., 2014). The cell walls of charophytes (streptophyte green algae) vary between taxonomic groups in a similar manner to the taxonomic group variation of land plants (Fangel *et al*., 2012). Polysaccharide‐based cell walls typical of land plants can be detected in Klebsormidiophyceae (Fig. 1), marking the transition from a wall centered on mineralized organic scales in the basal charophyte *Mesostigma viride* to one based on polysaccharides (Becker *et al*., 1991; Domozych *et al*., 1991; Sørensen *et al*., 2011). Cell wall complexity is found to increase in the more recently diverged classes, such as Coleochaetophyceae and Zygnematophyceae, seemingly presenting an all‐polysaccharide wall with the full complement of wall glycopolymers typical of plants, that is, cellulose, xylan, xyloglucan, mannan, mixed‐linkage glucan, and pectin. Hence, it has been proposed that what is often conceptualized as a ‘plant cell wall’, based on material composition and organization, emerged in a distant algal ancestor, most likely a common ancestor of Klebsormidiophyceae and plants (Sørensen *et al*., 2011; Mikkelsen *et al*., 2014).

Cellulose biosynthesis in plants is orthologous to that in prokaryotic organisms, but the origin of the biosynthetic activities for the remaining cell wall polysaccharides in higher plants is less clear. Xyloglucan and pectin putatively originated within charophyte algae (streptophyte green algae), while xylan, mannan, and mixed‐linkage glucan also exist in some noncharophyte algae and bacteria (Painter, 1983; Fangel *et al*., 2012; Salmeán *et al*., 2017). Hence, direct proof of functional orthology between algal and plant cell wall biosynthetic activities has been missing. This increases uncertainty as a result of the plausible convergent evolution of the different polymers, exemplified by the multiple occurrences of mixed‐linkage glucans throughout the tree of life. Identification of putative cell wall biosynthetic genes based on a *de novo* transcriptome assembly in charophytes has previously been published (Mikkelsen *et al*., 2014); however, this study utilized a limited transcriptome collection of insufficient quality, and carried out no biochemical confirmation of enzyme function, resulting in ambiguous conclusions. The completion of the *Klebsormidium flaccidum* genome sequence (Hori *et al*., 2014), in combination with the 1000 Plants (1KP ) Initiative (Matasci *et al*., 2014), now provides the opportunity to identify full‐length *K. flaccidum* gene sequences, resolve their phylogeny by comparison with algae and plants across the kingdom, and characterize the biochemical activities of gene products.

**Figure 2:**
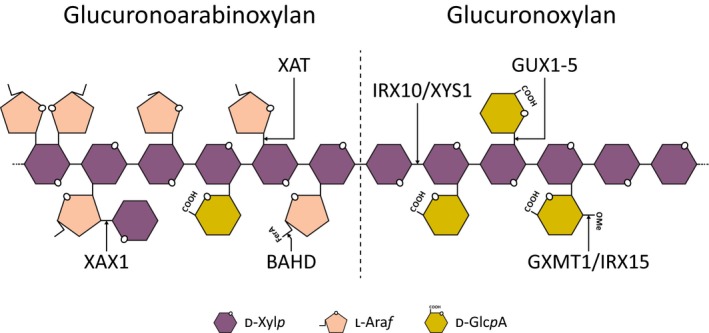
Schematic illustration of xylan structure with known biosynthetic activities represented.

Xylan is an abundant and complex cell wall component in plants, particularly in commelinid primary walls and in secondary cell walls of all angiosperms (Scheller & Ulvskov, 2010). It consists of a polymeric backbone of 1,4‐β‐linked d‐xylose (Xyl) decorated mainly with acetyl groups, and is further substituted by l‐arabino*furanose* (Ara*f*  ) in commelinids or methylated or unmethylated d‐glucuronic acid (GlcA) in noncommelinid angiosperms (Scheller & Ulvskov, 2010; Smith *et al*., 2017; Fig. 2). The elucidation of the fine structure of xylans found outside angiosperms is less complete and based only on a few species: gymnosperms produce methyl‐glucurono‐arabinoxylan (Busse‐Wicher *et al*., 2016), lycophytes and pteridophytes contain both methylated and unmethylated GlcA substitutions (Kulkarni *et al*., 2012), and the bryophyte *Physcomitrella patens* only contains unmethylated GlcA substituents (Kulkarni *et al*., 2012).

Proteins from eight protein families have been implicated in xylan biosynthesis, and based on their functions, 12 protein classes can be defined (Table 1; Fig. 2). For some of these classes, biochemical function has been established *in vitro* using recombinant proteins, including IRX10 and IRX10‐L, which are 1,4‐β‐xylan xylosyltransferases producing the 1,4‐β‐xylan backbone polymer (Jensen *et al*., 2014; Urbanowicz *et al*., 2014). In the case of IRX10‐L, long 1,4‐β‐xylan oligomers were produced *in vitro* and the protein was accordingly renamed XYLAN SYNTHASE‐1 (XYS1) (Urbanowicz *et al*., 2014). *P. patens* encodes one IRX10/XYS1 ortholog, *Pp*IRX10, which produces 1,4‐β‐xylan polymers that are of a similar size to those made by XYS1 *in vitro* (Jensen *et al*., 2014). Other putative glycosyltransferases from families GT8, GT43, and GT47 also affect xylan backbone formation when knocked out in *Arabidopsis*; however, their specific functions have remained unclear. Enzyme activities that contribute to 1,4‐β‐xylan backbone decorations include acetyl‐, methyl‐, and glycosyltransferases, and genes encoding the main enzyme activities have all been identified and characterized in angiosperms (Table 1).

**Table 1:** Phylogenetic origin of glycosyltransferases and accessory activities involved or putatively involved in xylan synthesis in plants

Xylan protein class	Protein family	Biochemical function	Earliest ortholog	Reference	Fig.
PARVUS	GT8	Unknown	Chlorophyta^a^	Brown *et al*. (2007)	S2
GXMT1/IRX15	DUF579	Glucuronoxylan methyltransferase/unknown	Chlorophyta^b^	Jensen *et al*. (2014); Urbanowicz *et al*. (2012)	S3
IRX10/XYS1	GT47	β‐1,4‐xylan synthase	Klebsormidiophyceae	Jensen *et al*. (2014); Urbanowicz *et al*. (2014)	3
IRX7	GT47	Unknown	Klebsormidiophyceae	Brown *et al*. (2007)	3
IRX9	GT43	Unknown	Klebsormidiophyceae	Brown *et al*. (2007)	S4
IRX14	GT43	Unknown	Klebsormidiophyceae	Brown *et al*. (2007)	S4
IRX8	GT8	Unknown	Klebsormidiophyceae	Peña *et al*. (2007)	S5
GUX1‐5	GT8	Xylan glucuronsyltransferase	Zygnematophyceae	Rennie *et al*. (2012)	S6
ESK1	DUF231	Acetylation	Unresolved, possibly angiosperms	Urbanowicz *et al*. (2014)	S7
XAT	GT61	Xylan arabinosyltransferases	Unresolved, possibly eudicot/monocot^c^	Anders *et al*. (2012)	S8
XAX1	GT61	Xylan xylosyltransferase	Unresolved, possibly Commelinid monocots	Chiniquy *et al*. (2012)	S8
BAHD	PF02458	Coumaric and ferulic acid transferases	Unresolved, possibly Liliales (noncommelinid monocot)	Bartley *et al*. (2013)	S9

a Chlorophyta orthologs were identified, but no charophyte algae and the earliest occurrence in plants was in Bryophyta.

b Chlorophyta and charophyte algae orthologs were identified ancestral to the split between IRX15(L) and GMXT1/GMXT2 clades.

c XAT orthologs have not been identified in Magnoliids or basal angiosperms.

Of the different plant cell wall polysaccharides, three in particular lend themselves to having their emergence in evolution pinpointed, that is, 1,4‐β‐xylan, xyloglucan, and the pectic polysaccharide homogalacturonan. We chose to focus on xylan and its biosynthesis in *K. flaccidum,* as this polymer was the earliest to emerge of the three and involves a number of biosynthetic activities that have been amenable to biochemical characterization (Sørensen *et al*., 2011; Rennie *et al*., 2012; Urbanowicz *et al*., 2012, 2014; Jensen *et al*., 2014). Our analysis points to the evolutionary appearance of the various xylan synthesis‐specific genes identified to date, describing innovations of this complex biocatalytic process as they occurred in two major phases over the course of plant evolution. Further, we demonstrated xylan synthase activity for one of the members of the earliest xylan synthesis‐specific gene homologs identified to date: a *K. flaccidum* IRX10/XYS1 ortholog (*Kf*XYS1). Finally, we identified and characterized the xylan in the cell walls of *K. flaccidum*, which is probably the product of the *Kf*XYS1. The enzymatic activity of *Kf*XYS1, combined with its evolutionary relatedness to modern IRX10/XYS1 xylan synthases, highlights the functional orthology between algal and plant cell wall biosynthesis.

## Materials and Methods

### Bioinformatics

The proteome files of the relevant divisions were obtained from the 1000 Plants (1KP) Initiative (Matasci *et al*., 2014). Relevant sequences for phylogenetic analysis from *P. patens*,* Selaginella moellendorffii*, Arabidopsis and rice were obtained using Harholt *et al*. (2012) as guide. A positive/negative list was made for identification of xylan‐related biosynthetic enzymes and the closest nonxylan related ortholog (Supporting Information Table S1). Sequences from this list were used as a database for blasting proteomes from the 1KP dataset as previously described (Mikkelsen *et al*., 2014), substituting the CAZy database with our positive list and eliminating false positives by substituting the Arabidopsis GT‐depleted database with our negative list. Phylogenetic analysis was performed as previously described (Ulvskov *et al*., 2013). Newick format tree files and sequences used in this manuscript are available in Notes S1. Evaluation of evolutionarily conserved protein motifs was performed using the SALAD website with their interactive service (Mihara *et al*., 2010; http://salad.dna.affrc.go.jp/salad/en/).

### Heterologous protein expression in *Saccharomyces cerevisiae*: purification and activity assays

For heterologous protein expression in *S. cerevisiae*,* YFP* was cloned into pESC‐URA by moving it from pPICZ A‐YFP (Jensen *et al*., 2014) using *Eco*RI and *Not*I. *KfXYS1* was obtained by *de novo* DNA synthesis (IDT, Coralville, IA, USA) with codon optimization for *S. cerevisiae* and cloned into the pESC‐URA‐YFP using *Eco*RI and *Pfl*MI restriction sites, generating pESC‐URA‐KfXYS1‐YFP. The sequence GTAATGGGT was engineered around the start codon for proper initiation of translation.

*Saccharomyces cerevisiae* YPH499 harboring pESC‐URA‐KfXYS1‐YFP was grown in SC synthetic minimal media (3.4 g Yeast Nitrogen Base l^−1^ media; ThermoFisher, Waltham, MA, USA), 2.8 g Yeast Synthetic Drop‐out Media (ThermoFisher), 10 g aluminum sulfate, 0.2 g leucine, 0.2 g tryptophan, 0.1 g histidine, and 0.2 g alanine plates with 2% glucose for 24 h at 30°C. Next, scrapes of these plates were used to inoculate 250 ml of liquid SC media with 2% glucose and incubated overnight in baffled flasks at 30°C in an orbital shaker at 180 rpm. At an OD_600_ of *c*. 2, the cells were collected by centrifugation (20 min at 2451 ***g***) and resuspended in SC media with 2% galactose, and then incubated at 18°C as before. Expression levels were monitored by fluorescent microscopy and cells were harvested by centrifugation 11 h after induction of recombinant protein expression. At the time of harvest, OD_600_ was *c*. 2, corresponding to *c*. 0.75 g of cells. Cell pellets were stored at −80°C. Protein purification and assays involving the fluorescently labeled xylooligosaccharide acceptor were performed as previously described (Jensen *et al*., 2014) and were based on *c*. 0.75 g of cells per batch.

### Heterologous protein expression and purification of *Kf*XYS1 in HEK293 cells

*Kf*XYS1 was cloned in a manner similar to that described in Urbanowicz *et al*. (2014). Briefly, to create Gateway entry clones, the truncated coding region of KfXYS1 (amino acids 27–445) was PCR‐amplified (KfXYS1_27F, 5′‐AACTTGTACTTTCAAGGCAGATCCTCTTTGTTCGT‐3′ and KfXYS1_445F, 5′‐ACAAGAAAGCTGGGTCCTAATTTTCATCATCACCACG‐3′) from pESC‐URA‐KfXYS1‐YFP plasmid DNA. A second set of universal primers (attB_Adapter‐F, 5′‐ GGGGACAAGTTTGTACAAAAAAGCAGGCTCTGAAAACTTGTACTTTCAAGGC‐3′ and attB_Adapter‐R, 5′‐GGGGACCACTTTGTACAAGAAAGCTGGGTC‐3′) was used to complete the *att*B recombination site and append a tobacco etch virus (TEV) protease cleavage site (Urbanowicz *et al*., 2014). The *att*B‐PCR product was cloned into the pDONR221 plasmid vector (Life Technologies, Carlsbad, CA, USA) using Gateway BP Clonase II Enzyme Mix (Life Technologies) to create an entry clone. To generate an expression clone of KfXYS1 (pGEn2‐EXP‐KfXYS1), the entry clone was recombined into a Gateway‐adapted version of the pGEn2 mammalian expression vector (pGEn2‐DEST) (Meng *et al*., 2013), using Gateway LR Clonase II Enzyme Mix (Life Technologies). The resulting expression construct (His‐GFP‐KfXYS1) encodes a fusion protein comprising an amino‐terminal signal sequence, an 8xHis tag, an AviTag recognition site, the ‘superfolder’ GFP (sfGFP) coding region, the recognition sequence of TEV protease, and residues 27–445 of *Kf*XYS1.

Recombinant expression and purification were performed by transient transfection of suspension culture HEK293‐F cells with pGEn2‐EXP‐KfXYS1 and a HisTap HP 1 ml column (GE Healthcare, Little Chalfont, UK), as previously described (Meng *et al*., 2013; Urbanowicz *et al*., 2014). Protein purity was assessed by sodium dodecyl sulfate‐polyacrylamide gel electrophoresis (Fig. S1). Purified His‐GFP‐KfXYS1 was concentrated to 0.45 mg ml^−1^ using an Amicon Ultra Centrifugal Filter Device (30000 MWCO; Merck Millipore; http://www.merckmillipore.com) and dialyzed (3500 MWCO) into HEPES sodium salt‐HCl (75 mM, pH 6.8) or sodium phosphate buffer (75 mM, pH 6.8) and used directly for reactions, or stored at 4 or −80°C in aliquots.

### MALDI‐TOF‐MS analysis of *Kf*XYS1 reaction products

Enzyme reactions (20 μl) consisted of 3 mM UDP‐xylose (UDP‐Xyl; Carbosource, Athens, GA, USA), 0.5 mM xylopentaose (Megazyme, Bray, Ireland), labeled at the reducing terminus with 2‐aminobenzamide as previously described (Ishii *et al*., 2002; Urbanowicz *et al*., 2014), and 4.5 μg of purified His‐GFP‐*Kf*XYS1 in HEPES sodium salt‐HCl buffer (75 mM, pH 6.8). Reactions were allowed to persist for 4 h before being prepared for analysis by matrix‐assisted laser desorption ionization‐time of flight mass spectrometry analysis on an LT Bruker LT Microflex spectrometer (Bruker, Billerica, MA, USA) as described previously (Urbanowicz *et al*., 2014). Positive‐ion spectra were recorded with a minimum of 200 laser shots summed to generate each spectrum.

### Nuclear magnetic resonance (NMR) analysis of *Kf*XYS1 reaction products

Products of a scaled‐up reaction were structurally characterized by NMR analysis. Reactions (300 μl) were carried out at 25°C in sodium phosphate buffer (75 mM, pH 6.8), containing 5 mM UDP‐Xyl, 1.5 mM xylobiose (Sigma) and 10 μg of purified His‐GFP‐KfXYS1. After 16 h, the reaction mixture was lyophilized, resuspended in 300 μl D_2_O (99.9%; Cambridge Isotope Laboratories, http://www.isotope.com) and characterized using a Varian 300 MHz NMR spectrometer (Varian, Palo Alto, CA, USA) at 25°C for initial characterization of the full reaction mixture composition. For characterization of higher‐order polysaccharide products only, the reaction mixture was fractionated on a Superdex 75 HR10/30 column and eluted with water to separate oligosaccharides and other reaction components. The fractions were lyophilized and resuspended in 200 μl D_2_O (99.9%; Cambridge Isotope Laboratories, Tewksbury, MA, USA). The 1D ^1^H NMR spectra were recorded at 25°C on a Varian Inova NMR spectrometer operating at 600 MHz and equipped with a 5 mm cold probe (Agilent, Santa Clara, CA, USA). Chemical shifts were measured relative to internal DMSO (δ^1^H 2.721) on both NMR instruments. Data were processed using MestReNova (Mestrelab Research, Santiago Compostela, Spain). Assignments for the UDP‐Xyl, UDP, xylobiose, and xylo‐oligosaccharides synthesized were made based on previously published results (Harper and Bar‐Peled, 2002; Peña *et al*., 2007; Wishart *et al*., 2009).

### Monosaccharide composition analysis

Cell wall material was prepared by grinding aliquots of 50 mg *K. flaccidum* cells in a TissueLyser MM 200 (Qiagen, Hilden, Germany) for 2 min at 30 s^−1^. Each sample was then extracted using 1.5 ml 70% ethanol for 5 d at 55°C, followed by four cycles of 1.5 ml 70% ethanol, one cycle of 1.5 ml acetone, and then air‐dried overnight. Monosaccharide composition was performed on cell wall material as previously described (Øbro *et al*., 2004) using a Dionex ICS 5000 + DC system (ThermoFisher) equipped with a  high‐performance anion exchange chromatograph with pulsed amperometric detection and a 4 μm SA‐10 column (2 × 250 mm and guard column). Run conditions were 40°C column temperature, 0.3 ml min^−1^ eluent flow rate, 1 mM NaOH for 0–8 min, followed by 100 mM NaOH from 8 to 20 min, and subsequently 10 min equilibration at 1 mM NaOH.

### Polysaccharide analysis using carbohydrate gel electrophoresis

Polysaccharide analysis using carbohydrate gel electrophoresis (PACE) was performed as previously described (Goubet *et al*., 2002) using GH10 xylanase (Brown *et al*., 2007), GH11 xylanase (Brown *et al*., 2011), GH115 α‐glucuronosidase (Rogowski *et al*., 2014), β‐xylosidase (Uniprot: Q92458) and arabinofuranosidase (NS39128). The last two enzymes were kindly provided by Novozymes (Novozymes, Bagsværd, Denmark).

### Comprehensive microarray polymer profiling

Samples of 10 mg *K. flaccidum* cell wall material were extracted with 300 μl of 50 mM diaminocyclohexanetetraacetic acid (CDTA; pH 7.5), and subsequently with 300 μl of 4 M NaOH with 0.1% w/w NaBH_4_. Extracts were spotted, probed and analyzed as previously described (Pedersen *et al*., 2012). Antibodies BS‐400‐2 (Meikle *et al*., 1991; Biosupplies, Bundoora, Australia), and LM10 (McCartney *et al*., 2005), LM11 (McCartney *et al*., 2005), LM12 (Pedersen *et al*., 2012), LM23 (Pedersen *et al*., 2012), LM27 (Cornuault *et al*., 2015) and LM28 (Cornuault *et al*., 2015; PlantProbes, Leeds, UK) were used for the analysis.

### Immunohistochemistry

*Klebsormidium flaccidum* cells were embedded in medium‐grade LR White (Polysciences, Hirschberg an der Bergstrasse, Germany) as previously described (Bell *et al*., 2013) and sectioned on a Reichert‐Jung/LKB Supernova ultramicrotome (2 μm; Reichert, Depew, NY, USA). Sections were treated with 5% skimmed milk in 100 mM PBS solution for 30 min and labeled using LM11 antibody (PlantProbes). A goat anti‐rat antibody conjugated to Alexa Fluor 555 was used to visualize the binding of LM11. Calcofluor White was applied to stain cell wall β‐glucans. Images were recorded on a Leica CLSM SP5 microscope (Leica, Heidelberg, Germany) using 405 nm excitation and 419–534 nm emission (Calcofluor White), and 543 nm excitation and 582–700 nm emission (Alexa Fluor 555). Negative controls using only the secondary antibody showed no nonspecific binding.

## Results

### Evolutionary appearance of genes involved in xylan biosynthesis

**Figure 3:**
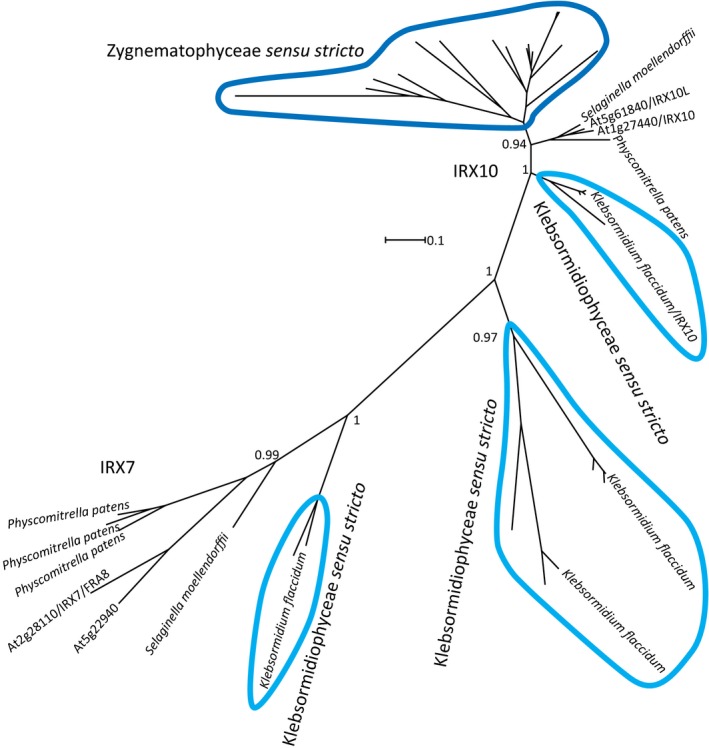
Phylogenetic tree of IRX10/XYS1 and IRX7 orthologs from charophytes, *Physcomitrella patens*,* Selaginella moellendorffii* and Arabidopsis. The *Klebsormidium flaccidum* xylan synthase is designated as *Kf* XYS1. No algal IRX10/XYS1 or IRX7 orthologs were identified outside Klebsormidiophyceae (light blue) or Zygnematophyceae (dark blue) as indicated by the *sensu stricto*. Sequences from Klebsormidiophyceae and Zygnematophyceae are ringed in light blue and dark blue, respectively. Branches with no name correspond to charophyte sequences from the 1KP dataset and are probably not full length. All branches with names are sequences from complete genome projects. Branch support was evaluated with an approximate likelihood ratio test and is given as a value between 0 and 1. Supporting Information Notes S1 holds tree files where all sequences used are labeled.

Utilizing the phylogenetic span of the 1KP dataset (Matasci *et al*., 2014) and genomic sequences from plants and *K. flaccidum* (Hori *et al*., 2014), we obtained phylogenetic trees with ample evolutionary width and resolution. These enabled us to study clade structures of protein families implicated in xylan biosynthesis, and to pinpoint the earliest orthologs for several of them (Table 1; Figs 3, S2–S9). These analyses suggest that xylan evolution occurred in two major phases, one in algae and a second in higher plants.

The early algal phase involves charophyte algae and the GT8, GT43, GT47 and DUF579/GXMT protein families. These represent candidate genes implicated in 1,4‐β‐xylan backbone formation, GlcA addition to the backbone, and subsequent GlcA methylation (Fig. 2). The second phase most probably involves flowering plants, specifically basal flowering plants and commelinid monocots, and the DUF231, GT61 and BAHD protein families (Fig. 2). Genes from these families have been implicated in xylan backbone acetylation, addition of arabinosyl and xylosyl substituents to the xylan backbone, and coumaric and ferulic acid transferase activity, respectively. Hence the second phase involves further diversification of xylan structure and probably reflects new functional role(s) of this glycopolymer in wall architecture.

The specific steps of the second phase of xylan evolution appear complex and remained unresolved. The DUF231, GT61 and BAHD protein families are each highly diversified in higher plants, and only a few members from each family have been linked to xylan synthesis. While ancient members of the families exist, none are closely related to the modern xylan‐specific members. Interestingly, we observed a high degree of diversification in basal flowering plants, and then later in commelinid monocots, particularly for GT61 and BAHD. One hypothesis is that xylan‐specific activities developed from different but related catalytic specificities. The time of diversification of these large protein families could in this way indicate the emergence of new specificities towards polymers such as xylan.

### A charophyte IRX10/XYS1 ortholog displays β‐1,4‐xylan synthase activity *in vitro*

Pinpointing early orthologs by sequence analysis primarily defines the oldest possible origin of the individual xylan‐specific activities. However, enzymatic activity of such orthologs is required to unambiguously determine if the modern activities are a result of catalytic conservation or if they evolved from a related enzyme that does not act in the xylan synthesis pathway. IRX10/XYS1 orthologs of the early algal phase are particularly interesting. While other proteins involved in 1,4‐β‐xylan backbone formation exist in plants, the IRX10/XYS1 orthologs are the only group of proteins that have been enzymatically implicated in 1,4‐β‐xylan backbone synthesis, and therefore play a central role among the proteins attributed to this process in plants.

**Figure 4:**
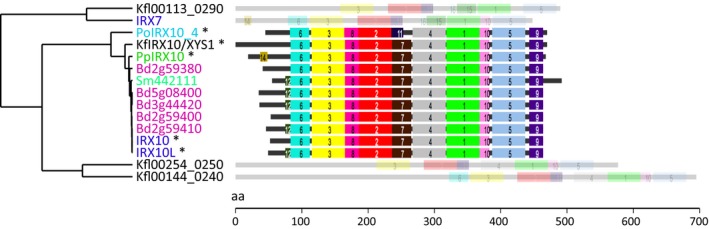
Protein motif analysis of the four IRX10/XYS1 and IRX7 orthologs from *Klebsormidium flaccidum*. Left part, hierarchical clustering of motif analysis. Right part, graphical representation of identified motifs. Proteins are aligned based on motif 9 (purple). If this motif is not present, the protein is shaded and based on the start codon. IRX10/XYS1 homologs from other plants include *Brachypodium distachyon* (pink), Arabidopsis (dark blue), *Plantago ovata* (light blue), *Selaginella moellendorffii* (light green), and *Physcomitrella patens* (dark green). Asterisks (*) indicate the five proven xylan xylosyltransferases/xylan synthase activities, including *Kf* XYS1. Analysis and representation were generated using the SALAD database (Mihara *et al*., 2010; http://salad.dna.affrc.go.jp/).

In *K. flaccidum*, four full‐length sequences showed sequence homology to IRX10/XYS1 and the closely related FRA8 (Table S2). Moreover, one of these proteins, designated *K. flaccidum* XYLAN SYNTHASE‐1 (*Kf*XYS1), showed a high degree of sequence conservation to IRX10/XYS1. blast protein alignment showed that *Kf*XYS1 and *At*XYS1 share a 0.75 identity score (75% amino acid sequence identity across 376 continuous residues of the 415 amino acid *At*XYS1 sequence; Table S3). This is a higher score than for the proven 1,4‐β‐xylan xylosyltransferase *Po*IRX10_4 from *Plantago ovata*, an herbaceous dicot that is phylogenetically much closer to Arabidopsis than to charophyte algae. In comparison, the *K. flaccidum* IRX8, IRX9 and IRX14 orthologs were all below 0.50 in identity scores with their respective Arabidopsis orthologs (Table S3), suggesting that these have been less well preserved. Furthermore, protein motif hierarchical clustering showed that all protein motifs shared among the four known 1,4‐β‐xylan xylosyltransferases (with the exception of a protein motif that is unique to *Po*IRX10_4) are conserved in *Kf*XYS1 (Fig. 4).

**Figure 5:**
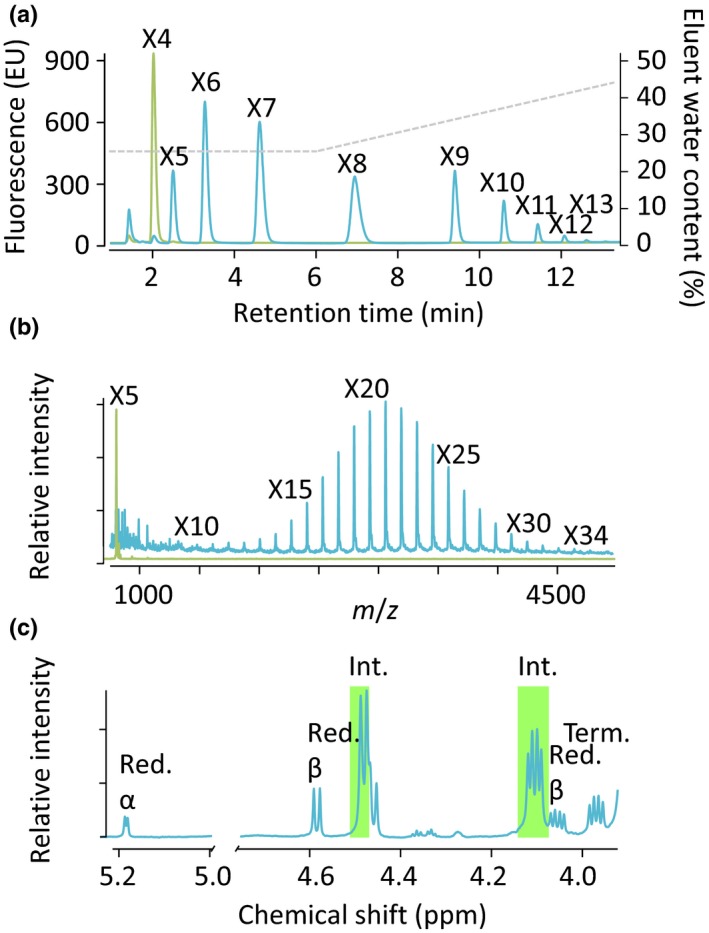
Xylan synthase activity of purified *Kf* XYS1 expressed in two different heterologous expression systems. (a) Xylan synthase assay using purified *Kf* XYS1‐YFP produced in *Saccharomyces cerevisiae*. high‐performance liquid chromatography analysis of reaction mixtures comprising UDP‐xylose and fluorescently labeled xylotetraose (X4) incubated in the presence (blue) or absence (green) of *Kf* XYS1‐YFP. The gray dashed line represents eluent water content (right *y*‐axis). X4‐X13 designate progressively longer xylan oligosaccharides. (b) Xylan synthase assay using purified His‐GFP‐*Kf* XYS1 produced in HEK293 cells. Matrix‐assisted laser desorption ionization‐time of flight mass spectrometry of reaction mixtures after a 4 h reaction in the absence (green) and presence (blue) of UDP‐xylose. The series of annotated [M + H]^+^ ions are the result of structures with a mass difference of 132 Da, consistent with the sequential addition of xylosyl residues to the 2‐aminobenzamide‐labeled xylopentaose acceptor (X5) generating oligosaccharides with a degree of polymerization (DP) up to 34 (X34). (c) 1D ^1^H nuclear magnetic resonance (NMR) analysis of reaction products generated by incubation of His‐GFP‐*Kf* XYS1 with UDP‐xylose as a donor and unlabeled xylobiose as an acceptor substrate. After a 16 h reaction time, xylobiose, UDP‐xylose, and UDP were separated away from higher‐order oligosaccharide products by size exclusion chromatography before NMR analysis. The spectrum shows diagnostic signals of internal β‐(1,4)‐linked xylosyl residues (Int., green shade; Red, Reducing; Term, Terminal).

A high degree of conservation is supportive of xylan synthase activity; however, to provide biochemical evidence we heterologously expressed *Kf*XYS1 and characterized its enzymatic activity. Initially, we expressed *Kf*XYS1 in *S. cerevisiae* with a C‐terminal YFP protein tag to facilitate affinity purification. Purified *Kf*XYS1‐YFP was incubated in the presence of UDP‐Xyl and 1,4‐β‐xylotetraose fluorescently labeled with anthranilic acid, and the reaction products were analyzed by normal‐phase high‐performance liquid chromatography. Xylan xylosyltransferase activity was evident by the appearance of multiple new peaks, relative to the control containing only 1,4‐β‐xylotetraose (Fig. 5a). These data suggested that up to nine successive Xyl transfer events occurred under these conditions. Activity levels and peak retention times are comparable to reactions previously reported for *At*XYS1 and *Pp*IRX10 (Jensen *et al*., 2014; Urbanowicz *et al*., 2014). A partial digestion of the *Kf*XYS1‐YFP reaction products with a 1,4‐β‐xylan‐specific xylanase further supported the idea that the linkages catalyzed by *Kf*XYS1 are indeed β‐1,4‐Xyl linkages (Fig. S10a). No activity was detected when using UDP‐glucose, UDP‐arabino*pyranose* (UDP‐Ara*p*) or UDP‐arabino*furanose* (UDP‐Ara*f*) as donor substrates (Fig. S10b).

Xylan polymers consisting solely of 1,3‐β‐Xyl linkages and mixed‐linkage (1,3)(1,4)‐β‐xylans occur in chlorophytes and red algae (Painter, 1983). To further investigate the linkage composition of the *Kf*XYS1 reaction products by NMR spectroscopy, we heterologously expressed the protein in HEK293 cells. A predicted transmembrane domain in the N‐terminus of KfXYS1 was substituted with a His‐GFP protein tag in the HEK293 heterologous expression system, and the recombinant *Kf*XYS1 protein was expressed and purified (His‐GFP‐*Kf*XYS1). Expression and secretion of the His‐GFP‐*Kf*XYS1 fusion protein in transiently transfected HEK293F cells resulted in high amounts of enzyme secretion (*c*. 100 mg l^−1^) as determined by measuring the relative fluorescence (GFP fluorescence ^His‐GFP‐*Kf* XYS1^, 1380) of the recombinant protein secreted into the media (Meng *et al*., 2013; Urbanowicz *et al*., 2017), facilitating the large‐scale reactions suitable for detailed product analyses. Expression in HEK293F cells resulted in highly active enzyme preparations capable of adding as many as 29 Xyl residues to a starting 2‐aminobenzamide‐xylopentaose acceptor (Fig. 5b). 1D ^1^H NMR characterization of reaction mixtures containing xylobiose, UDP‐Xyl and His‐GFP‐*Kf*XYS1 showed depletion of UDP‐Xyl and an increase in internal 1,4‐β‐Xyl linkages, relative to control reactions containing only xylobiose and UDP‐Xyl (Fig. S11). To obtain a less complex spectrum derived from only the higher‐order polysaccharide products, the reaction mixture was subjected to size exclusion chromatography to separate these from other reaction constituents, including UDP and xylobiose. This 1D ^1^H NMR spectrum revealed prominent diagnostic signals of internal 1,4‐β‐Xyl linkages (Urbanowicz *et al*., 2014) (Fig. 5c), while 1,3‐β‐Xyl linkages, which would produce a clear signal at 4.68 ppm (Viana *et al*., 2011), were found to be absent.

These results show that *Kf* XYS1 expressed in two separate heterologous expression systems using both N‐ and C‐terminal protein tagging, displays 1,4‐β‐xylan synthase activity exclusively, consistent with a high level of protein homology to known plant xylan synthases. Hence, the enzymatic activity of this group of proteins has been conserved as far back as an extinct common ancestor of *K. flaccidum* and land plants, living *c*. 700 million yr ago (Douzery *et al*., 2004).

### *Klebsormidium flaccidum* cell walls contain multiple species of substituted xylan

**Figure 6:**
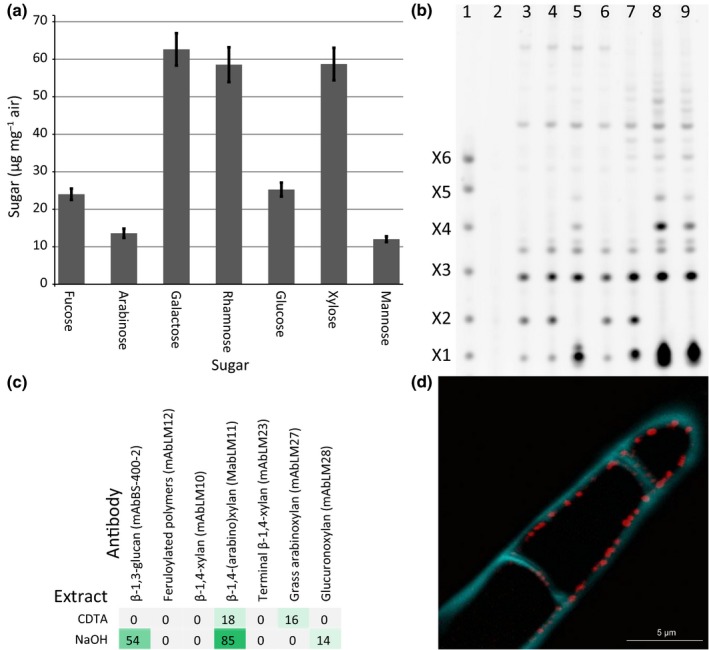
Structural analysis of *Klebsormidium flaccidum* cell walls. (a) Monosaccharide composition of *K. flaccidum* cell wall material (*n *=* *3, ± SD). (b) Cell wall material analyzed by polysaccharide analysis using carbohydrate gel electrophoresis using hydrolytic enzymes specific for the 1,4‐β‐xylan backbone or side chain modifications. Lane 1, marker lane, 1,4‐β‐xylan oligosaccharides with degree of polymerization 1 to 6; lane 2, control digestion with no enzyme; lane 3, GH10 1,4‐β‐xylanase; lane 4, GH10 and GH11 1,4‐β‐xylanases; lane 5, GH10 and xylosidase; lane 6, GH10 and GH115 glucuronidase; lane 7, GH10 xylanase plus GH43 and GH62 arabinofuranosidases; lane 8, as lane 7 plus 1,4‐β‐xylosidase and GH115; lane 9, as lane 8 plus GH11. (c) Sequential extraction of *K. flaccidum* cell wall material and relative quantification of cell wall epitopes by comprehensive microarray polymer profiling (CoMPP). The values in the heat map are mean spot signals from three experiments and the highest signal in the entire dataset was set to 100 and all other data were adjusted accordingly. A cutoff value of 5 was imposed. (d) Immunolocalization of xylan (antibody LM11; red) and β‐glucan (Calcofluor White; blue) in the *K. flaccidum* cell wall by confocal laser scanning microscopy.

Having determined that *Kf*XYS1 is a β‐1,4‐xylosyltransferase, we analyzed *K. flaccidum* cell walls to determine whether they contain 1,4‐β‐xylan. Monosaccharide compositional analysis of *K. flaccidum* cell wall material showed that they contain 59 μg Xyl mg^−1^ cell wall material, making it one of the dominant monosaccharide constituents (Fig. 6a). To specifically confirm the presence of 1,4‐β‐xylan and to characterize its structure, we extracted *K. flaccidum* cell wall material with strong base and subjected these to 1,4‐β‐xylanase digestion. The resulting xylan oligosaccharides were analyzed by gel electrophoresis (PACE). Digestion with GH10 1,4‐β‐xylanase, which requires one unsubstituted Xyl residue for cleavage, resulted in a complex band pattern, indicating the presence of 1,4‐β‐xylan with frequent substitutions. Two bands comigrated with either Xyl or 1,4‐β‐xylobiose (Fig. 6b, lane 3). Subsequent digestion with GH3 β‐xylosidase resulted in a depletion of the xylobiose band and an increase in the Xyl band, confirming their individual identities (Fig. 6b, lane 5). The oligosaccharide migrating near xylotriose was insensitive to β‐xylosidase, indicating that it is not xylotriose, but probably a substituted oligosaccharide. The xylo‐oligosaccharides were insensitive to digestion with GH115 α‐glucuronosidase (Fig. 6b, lanes 6, 8 and 9). By contrast, digestion with diagnostic xylan α‐arabinofuranosidases (GH43 and GH62) resulted in alterations in band patterning, indicating the presence of α‐Ara*f* substituents (Fig. 6b, lane 7–9). The observation that not all the bands were fully digested by these enzymes suggests that there are other kinds of substitutions present in *K. flaccidum* xylan and possibly linkages other than 1,4‐β‐Xyl in the backbone. In conclusion, *K. flaccidum* cell wall material shows diagnostic band patterns of 1,4‐β‐xylan with α‐linked arabinosyl substitutions and other additional unidentified modifications or linkages in the backbone. Phylogenetic analysis of the known arabinosyltransferase from GT61 indicated that no orthologs are present in *K. flaccidum* (Table 1). Therefore, this suggests that another class of glycosyltransferases carries out this function in *K. flaccidum*, representing a case of convergent evolution with regard to 1,4‐β‐xylan α‐arabinosyl decorations.

Sequential extraction of *K. flaccidum* cell wall material with subsequent immobilization to nitrocellulose membranes and probing with xylan‐directed antibodies (comprehensive microarray polymer profiling) resulted in substantial labeling by the 1,4‐β‐xylan‐specific antibody LM11 in both the CDTA and the NaOH‐soluble fractions (Fig. 6c). Additionally, by employing antibodies directed at xylan with complex substitution patterns (LM27, binding to xylan with complex substitution patterns such as grass xylan, and LM28, binding to methylated and unmethylated glucuronosyl substituted xylan) we were able to confirm the complex substitution pattern suggested by the PACE analysis. LM27 and LM28 epitopes were present in separate fractions, suggesting that at least two structurally different pools of xylan are present in *K. flaccidum*. Immunohistochemical localization with a xylan‐specific antibody (LM11) showed that xylan was present in the *K. flaccidum* cell wall in a punctate but distinct pattern on the luminal side of the cell, which may be interpreted to be labeling of newly synthesized xylan immediately before integration into the wall matrix (Fig. 6d).

## Discussion

The evolution of a polysaccharide‐based cell wall was probably a key event facilitating terrestrialization, making this a defining moment in the evolution of life on land. A growing body of evidence suggests that charophyte algae were the first to inhabit land, and from these algae, land plants subsequently evolved *c*. 475 million yr ago (Harholt *et al*., 2016). In the present study, we show enzymatic conservation between 1,4‐β‐xylan synthases separated by *c*. 700 million yr of evolution. Interestingly, our data‐mining efforts did not identify any additional homologs predating *Kf*XYS1. Using specific antibodies and hydrolytic enzymes, we also showed that *K. flaccidum* cell walls contain substituted 1,4‐β‐xylans. These xylans are probably the products of a pathway involving *Kf*XYS1. Our study therefore suggests that charophyte algae are the evolutionary origin of the 1,4‐β‐xylans of modern‐day plants.

The xylan synthase activity of *Kf*XYS1 is pivotal evidence in support of the hypothesis that the ‘plant cell wall’ emerged in charophyte algae. For instance, the biochemical orthology between *Kf*XYS1 and embryophyte IRX10/XYS1 makes it likely that other seemingly functionally orthologous relationships in xyloglucan and pectin biosynthesis between charophyte algae and land plants are indeed genuine. With established functional orthology between early diverging charophyte alga and land plant cell wall biosynthetic processes, we can now establish with reasonable certainty that the land plant cell wall originated in an early ancestor in charophyte algae evolution.

Interestingly, IRX9 and IRX14, which are glycosyltransferase‐like proteins required for xylan synthesis *in planta*, also appear in Klebsormidiophyceae (Figs 2, S4). These proteins do not seem to have essential catalytic functions in higher plants, and most likely have a role in anchoring IRX10 in the Golgi apparatus, as observed in Arabidopsis (Ren *et al*., 2014) and asparagus (Zeng *et al*., 2016). The function of IRX9 and IRX14 in Klebsormidiophyceae could be the same as in these higher plants, or they may be unrelated and were recruited later in evolution to participate in xylan synthesis. PARVUS and GXMT1 provide examples of the latter scenario as they are both implicated in xylan biosynthesis in Arabidopsis yet have orthologs in chlorophytes that do not produce xylan.

*Klebsormidium flaccidum* xylan appears to be highly substituted, but no GT61 or GUX orthologs were identified in *K. flaccidum* (Table 1; Fig. 2). This suggests that other classes of glycosyltransferases are involved in decorating its xylan backbone. This includes the apparent α‐arabinosyl substituents, which have been associated with GT61 enzymes in grasses (Anders *et al*., 2012), suggesting a case of convergent evolution. Other examples exist where backbone substitutions are apparently reinvented readily, for example in xyloglucan side chain evolution (Tuomivaara *et al*., 2015) and possibly in psyllium seed mucilaginous layers that produce a highly arabinosyl‐ and xylosyl‐substituted heteroxylan and encode an unusually high number of GT61 orthologs (Jensen *et al*., 2013).

While no GUX orthologs were identified in the *K. flaccidum* genome (consistent with its xylan being insensitive to α‐glucuronosidase and no observable GlcA in the sugar composition analysis), we identified an ortholog in Zygnematophyceae, the most recent group of charophyte algae to have evolved (Fig. 1). The GUX proteins form three phylogenetic subgroups, that is, charophytes, mosses and vascular plants (Fig. S6), with glucuronoxylan isolated and characterized from the latter two subgroups of plants (Kulkarni *et al*., 2012; Rennie & Scheller, 2014; Busse‐Wicher *et al*., 2016). This suggests that the GUX proteins function as xylan glucuronosyltransferases in mosses. The vascular plant GUX subgroup further shows division between subgroups of Arabidopsis GUX2/4/5 and GUX1/3 orthologs. In Arabidopsis, even spacing of GlcA residues along the xylan backbone in secondary cell walls is provided by GUX1 (Bromley *et al*., 2013) and in primary cell walls by GUX3 (Mortimer *et al*., 2015). The even GlcA spacing facilitates binding to cellulose microfibrils through a twofold helical screw ribbon conformation of the glucuronoxylan, which probably exerts an influence on microfibril aggregation and organization (Simmons *et al*., 2016). The phylogenetic analysis therefore suggests that this prominent architectural principle arose in basal vascular plants.

As we see the contours of a polysaccharide‐based cell wall evolve in charophytic algae, and its further evolution throughout the plant kingdom, a number of questions become increasingly pressing. Why were these polysaccharide classes (i.e. cellulose, xylan, xyloglucan, mannan, and pectin) selected? Why are some occasionally expendable in some cell walls and cell types, while seemingly indispensable to the organism as a whole and maintained consistently in land plants throughout evolution? Why have so few new polysaccharide classes, such as Rhamnogalacturonan II, emerged during the course of plant evolution? Why does utilization of different polysaccharides change during evolution and what differences in cell wall functionality, architectural principles and solutions drive these evolutionary events? By having a firmer understanding of the evolutionary span and context of the plant cell wall, we are now poised to begin to answer these fundamental questions in plant biology.

In conclusion, using a multifaceted approach involving genome and transcriptome mining and phylogenetic and biochemical analysis, *Kf* XYS1 was identified and shown to possess 1,4‐β‐xylan synthase activity. Immunolabeling and biochemical analysis of *K. flaccidum* cell walls identified a cell wall‐localized, highly substituted 1,4‐β‐xylan. These findings, along with an evolutionary analysis of the occurrence of xylan pathway enzymes, link plant xylan evolution to an ancestral charophytic alga with a diverging point *c*. 700 million yr ago.

## Author contributions

J.K.J. and J.H. conceived and initiated the project. J.K.J., M.B‐W., C.P.P., H.J.M., P.J.S., B.R.U. and J.H. designed experiments. J.K.J., M.B‐W., C.P.P., J.U.F., M.H.D., H.J.M., K.W.M., P.J.S., B.R.U., J‐Y.Y., M‐J.P., C.G.W., P.D., H.V.S., P.U. and J.H. performed experiments and interpreted the results. M.M. and G.K‐S.W. provided unpublished sequences. The manuscript was written by J.K.J., B.R.U. and J.H. All authors edited and commented on the manuscript.

## Supporting information

Please note: Wiley Blackwell are not responsible for the content or functionality of any Supporting Information supplied by the authors. Any queries (other than missing material) should be directed to the *New Phytologist* Central Office.

**Fig. S1** SDS gel of purified GFP‐KfXYS1 expressed in HEK293 cell culture.
**Fig. S2** Phylogenetic tree of PARVUS orthologs.
**Fig. S3** Phylogenetic tree of IRX15/GXMT1 orthologs.
**Fig. S4** Phylogenetic tree of IRX9 and IRX14 orthologs.
**Fig. S5** Phylogenetic tree of IRX8 orthologs.
**Fig. S6** Phylogenetic tree of GUX1‐5 orthologs.
**Fig. S7** Phylogenetic tree of ESK1 orthologs.
**Fig. S8** Phylogenetic tree of GT61 orthologs.
**Fig. S9** Phylogenetic tree of *Os*AT10 orthologs.
**Fig. S10** Enzymatic specificity of *KfXYS1*.
**Fig. S11** Characterization of reaction mixtures with or without *Kf*XYS1 by 1D ^1^H NMR.
**Table S1** Positive/negative list used for identification of xylan synthesis‐related sequences
**Table S2** Protein sequences of *Klebsormidium flaccidum* xylan gene orthologs
**Table S3** Protein blast analysis of *Klebsormidium flaccidum* xylan gene orthologs

**Notes S1** Newick tree files and sequences used for Figs 2 and S1–S8.
