# Supplementary material for: Identification of an algal xylan synthase indicates that there is functional orthology between algal and plant cell wall biosynthesis
Source: New Phytol. 2018 Feb 20;218(3):1049–60. doi: 10.1111/nph.15050 (PMC5902652; doi:10.1111/nph.15050)
Supplement: Supplementary file 1 — Fig. S1 SDS gel of purified GFP‐KfXYS1 expressed in HEK293 cell culture. Fig. S2 Phylogenetic tree of PARVUS orthologs. Fig. S3 Phylogenetic tree of IRX15/GXMT1 orthologs. Fig. S4 Phylogenetic tree of IRX9 and IRX14 orthologs. Fig. S5 Phylogenetic tree of IRX8 orthologs. Fig. S6 Phylogenetic tree of GUX1‐5 orthologs. Fig. S7 Phylogenetic tree of ESK1 orthologs. Fig. S8 Phylogenetic tree of GT61 orthologs. Fig. S9 Phylogenetic tree of OsAT10 orthologs. Fig. S10 Enzymatic specificity of KfXYS1. Fig. S11 Characterization of reaction mixtures with or without KfXYS1 by 1D 1H NMR. Table S1 Positive/negative list used for identification of xylan synthesis‐related sequences Table S2 Protein sequences of Klebsormidium flaccidum xylan gene orthologs Table S3 Protein blast analysis of Klebsormidium flaccidum xylan gene orthologs [file NPH-218-1049-s001.pdf]

***New Phytologist* Supporting Information**

Article title: Identification of an algal xylan synthase KfXYS1 proves that there is functional orthology between algal and plant cell wall biosynthesis
Authors: Jacob Krüger Jensen, Marta Busse-Wicher, Christian Peter Poulsen, Jonatan Ulrik Fangel, Peter James Smith, Jeong-Yeh Yang, Maria-Jesus Peña, Malene Hessellund Dinesen, Helle Juel Martens, Michael Melkonian, Gane Ka-Shu Wong, Kelley W. Moremen, Curtis Gene Wilkerson, Henrik Vibe Scheller, Paul Dupree, Peter Ulvskov, Breeanna Rae Urbanowicz and Jesper Harholt
Article acceptance date: 14 January 2018

The following Supporting Information is available for this article:

Fig. S1 SDS-gel of purified GFP-KfXYS1 expressed in HEK293 cell culture

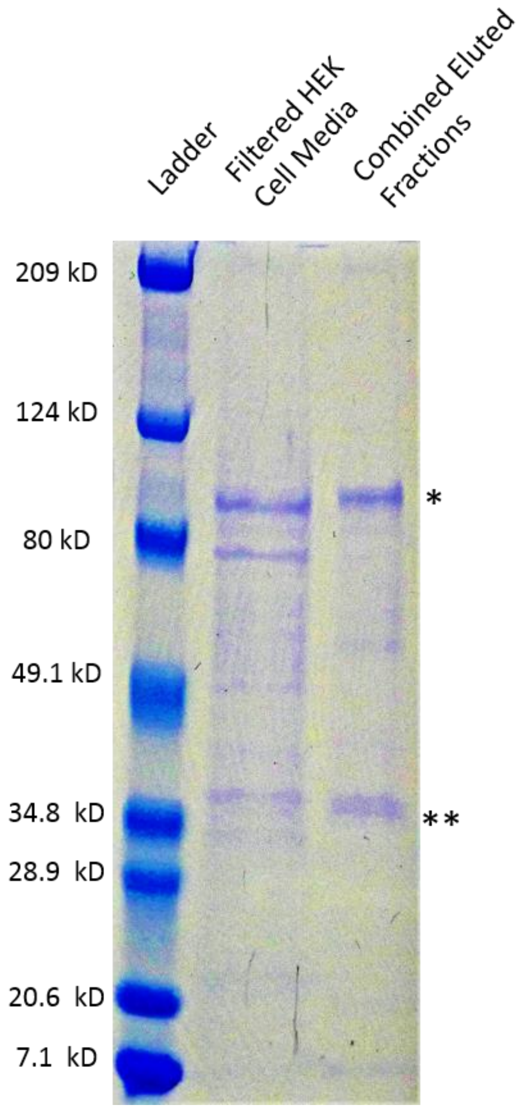

Fig. S1 Purification of recombinant GFP-KfXYS1. Lane 1, Broad Range Prestained SDS-PAGE Standard (Bio-Rad); Lane 2, HEK293 cell culture medium; Lane 3, pooled eluted protein. The gel was stained with coomassie brilliant blue R-250 (Bio-Rad). *KfXYS1(MW 85.46kD) and **His-Avi-GFP (MW 33.2kD).

Fig. S2 Phylogenetic tree of PARVUS orthologs.

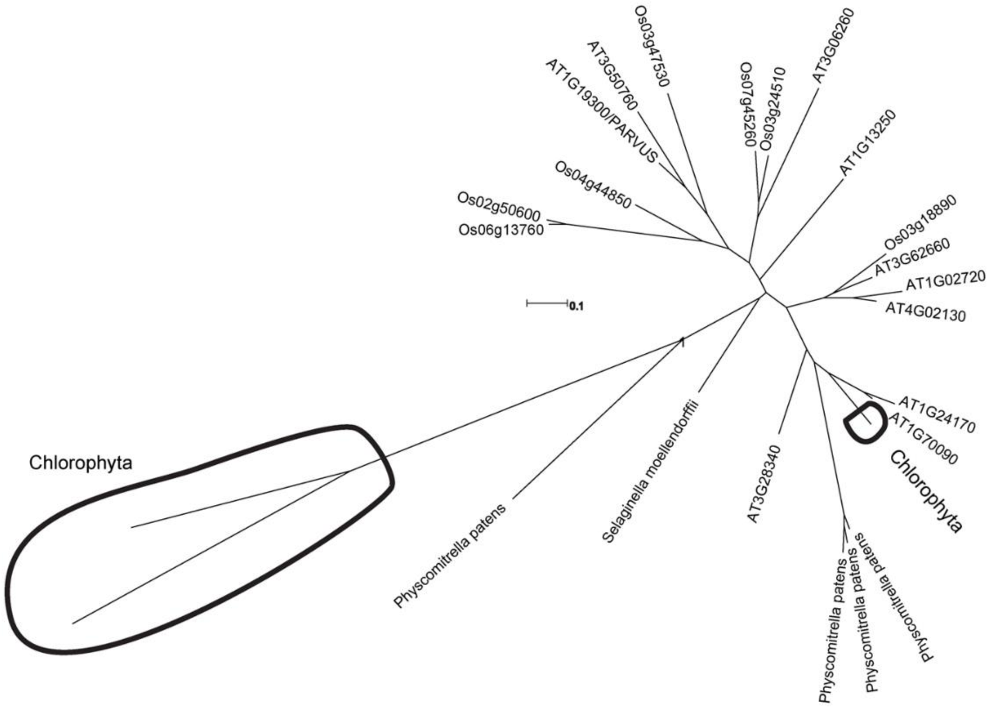

Fig. S2. Phylogenetic tree of PARVUS orthologs. Sequences included from chlorophytes, *Physcomitrella patens*, *Selaginella mollendorffii*, rice and Arabidopsis. The two encirclings indicate sequences from chlorophytes (black). Branches with no name correspond to chlorophyte sequences from 1kp dataset and are probably not full length. All branches with names are sequences from complete genome projects. Branch support was evaluated with Approximate Likelihood-Ratio Test and is given as a value between 0 and 1. Supporting Information Notes S1 holds tree files where all sequences used are labeled.

Fig. S3 Phylogenetic tree of IRX15/GXMT1 orthologs.

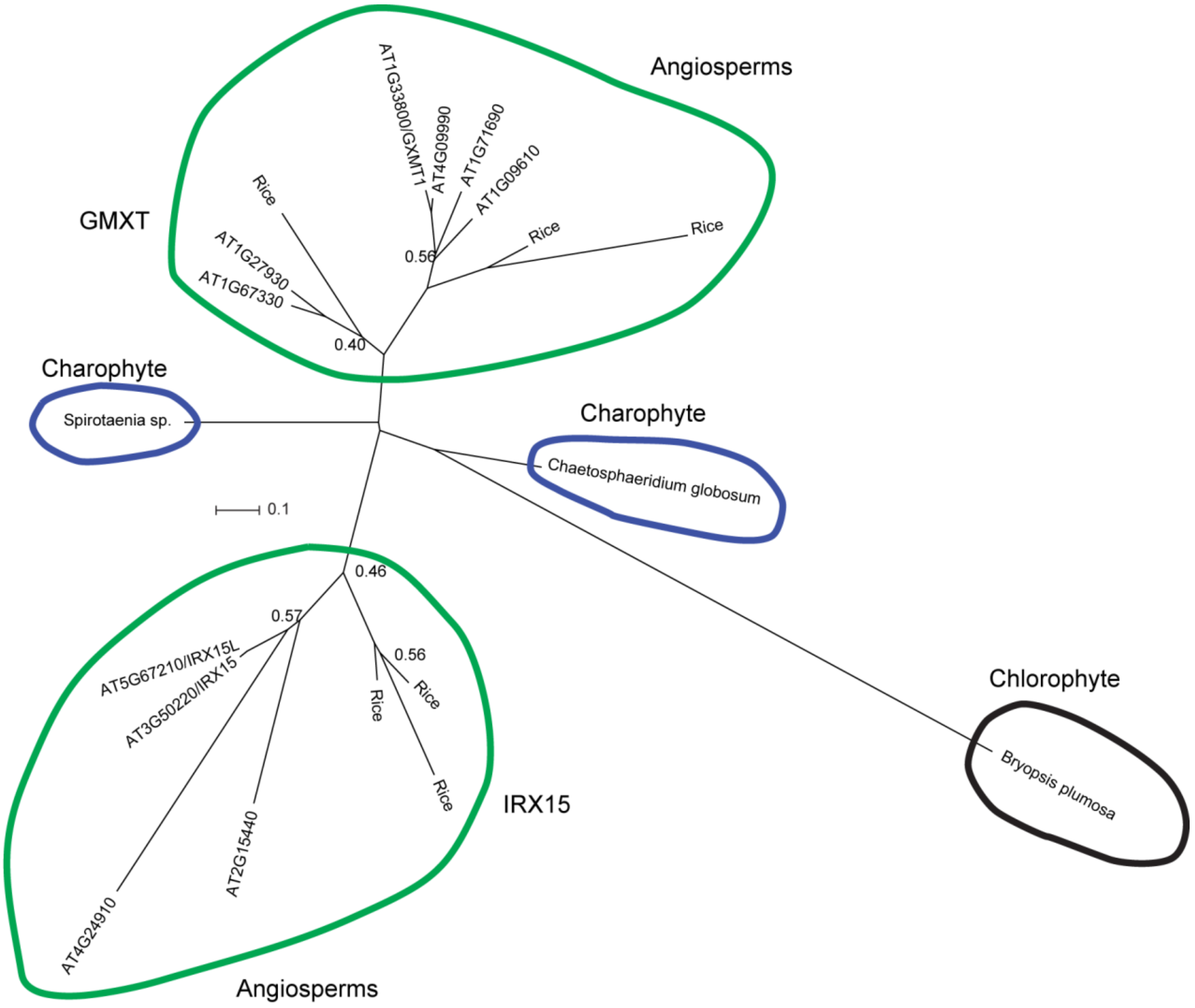

Fig. S3. Phylogenetic tree of IRX15/GXMT1 orthologs. Sequences included from chlorophyte and charophyte green algae, rice and Arabidopsis. Encircling indicate sequences from angiosperms (green), chlorophytes (black) and charophytes (dark blue). For angiosperms, the IRX15 and GXMT1 orthologs group in two separate groups, respectively. Chlorophyte and charophyte branches correspond to sequences from 1kp dataset and are probably not full length. Branch support was evaluated with Approximate Likelihood-Ratio Test and is given as a value between 0 and 1. Supporting Information Notes S1 holds tree files where all sequences used are labeled.

Fig. S4 Phylogenetic tree of IRX9 and IRX14 orthologs.

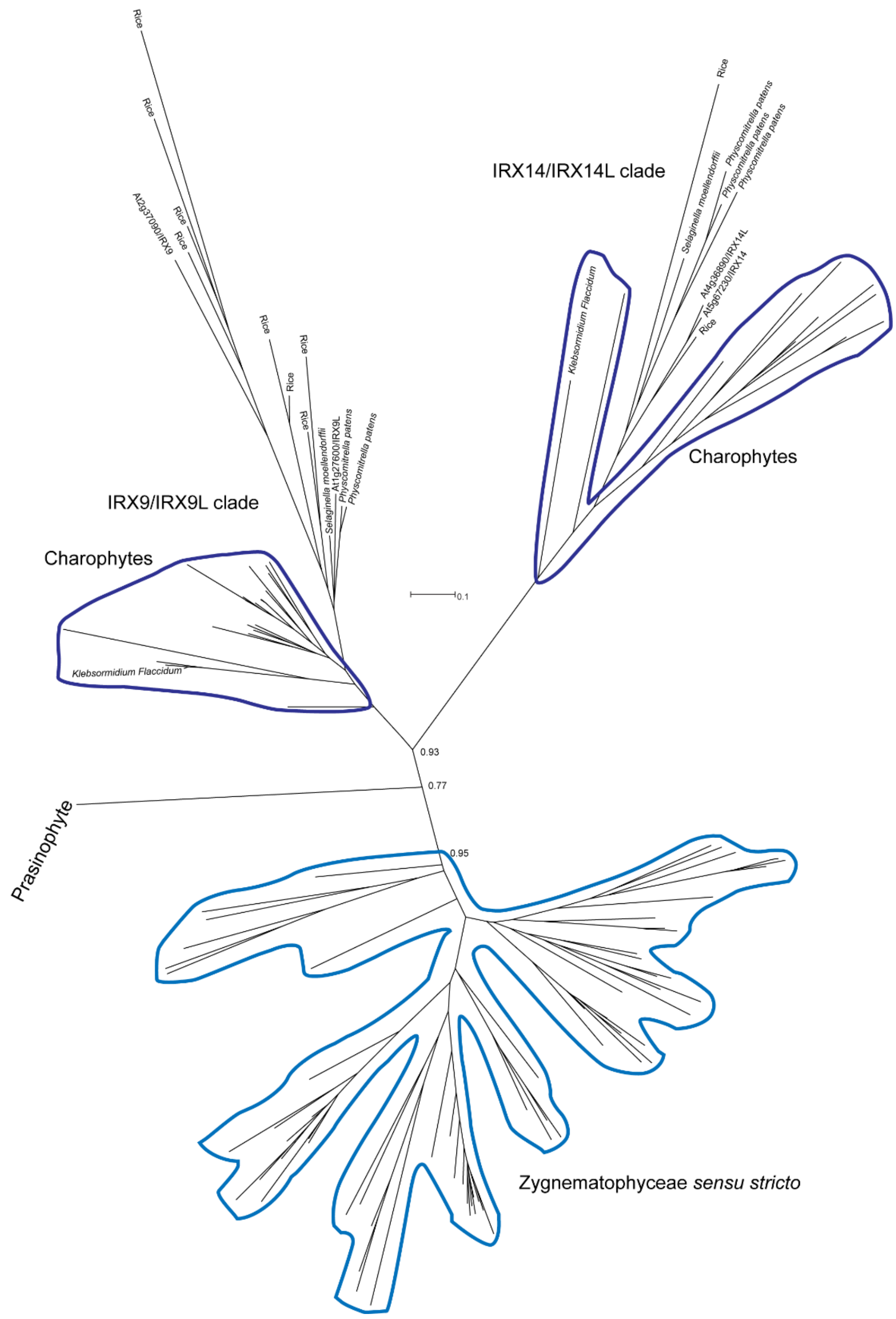

Fig. S4. Phylogenetic tree of IRX9 and IRX14 orthologs. Sequences included from *Ostreococcus lucimarinus* (prasinophyte), charophytes, *Klebsormidium flaccidum*, *Physcomitrella patens*,

Selaginella moellendorffii, rice and Arabidopsis. Encircling indicate charophytes (dark blue) and Zygnematophyceae sensu stricto (light blue). Branches with no name correspond to charophyte sequences from 1kp dataset and are probably not full length. All branches with names are sequences from complete genome projects. Branch support was evaluated with Approximate Likelihood-Ratio Test and is given as a value between 0 and 1. Supporting Information Notes S1 holds tree files where all sequences used are labeled.

Fig. S5 Phylogenetic tree of IRX8 orthologs.

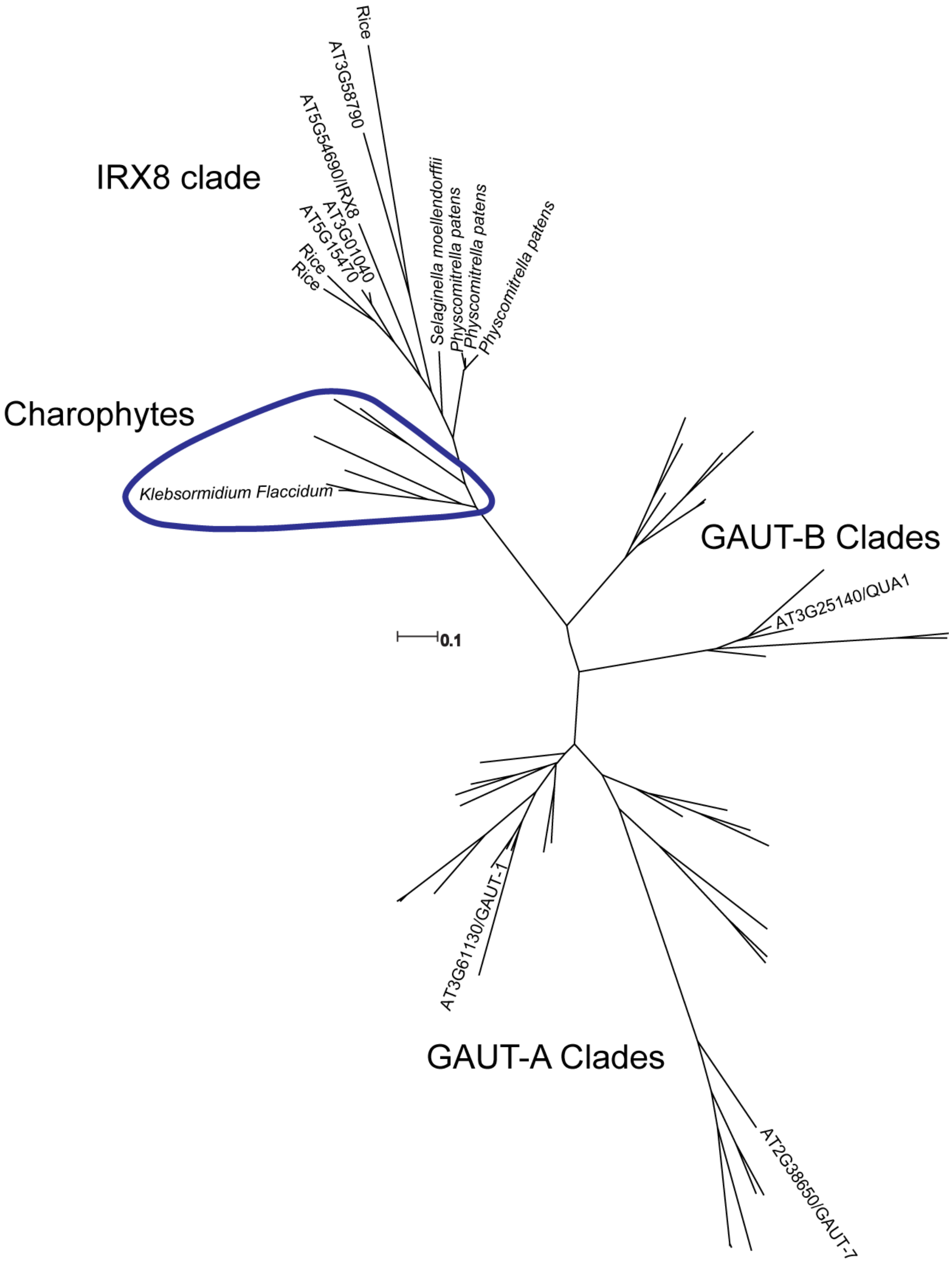

Fig. S5. Phylogenetic tree of IRX8 orthologs. Sequences included from charophytes, *Klebsormidium flaccidum*, *Physcomitrella patens*, *Selaginella moellendorffii*, rice and Arabidopsis. Encircling indicates sequences from a group of several species of charophytes

(dark blue). Branches in encircling with no name correspond to charophyte sequences from 1kp dataset and are probably not full length. Branches in GAUT-A and GAUT-B clades are full length sequences from *Physcomitrella patens*, *Selaginella moellendorffii*, rice and Arabidopsis. Branch support was evaluated with Approximate Likelihood-Ratio Test and is given as a value between 0 and 1. Supporting Information Notes S1 holds tree files where all sequences used are labeled.

Fig. S6 Phylogenetic tree of GUX1-5 orthologs.

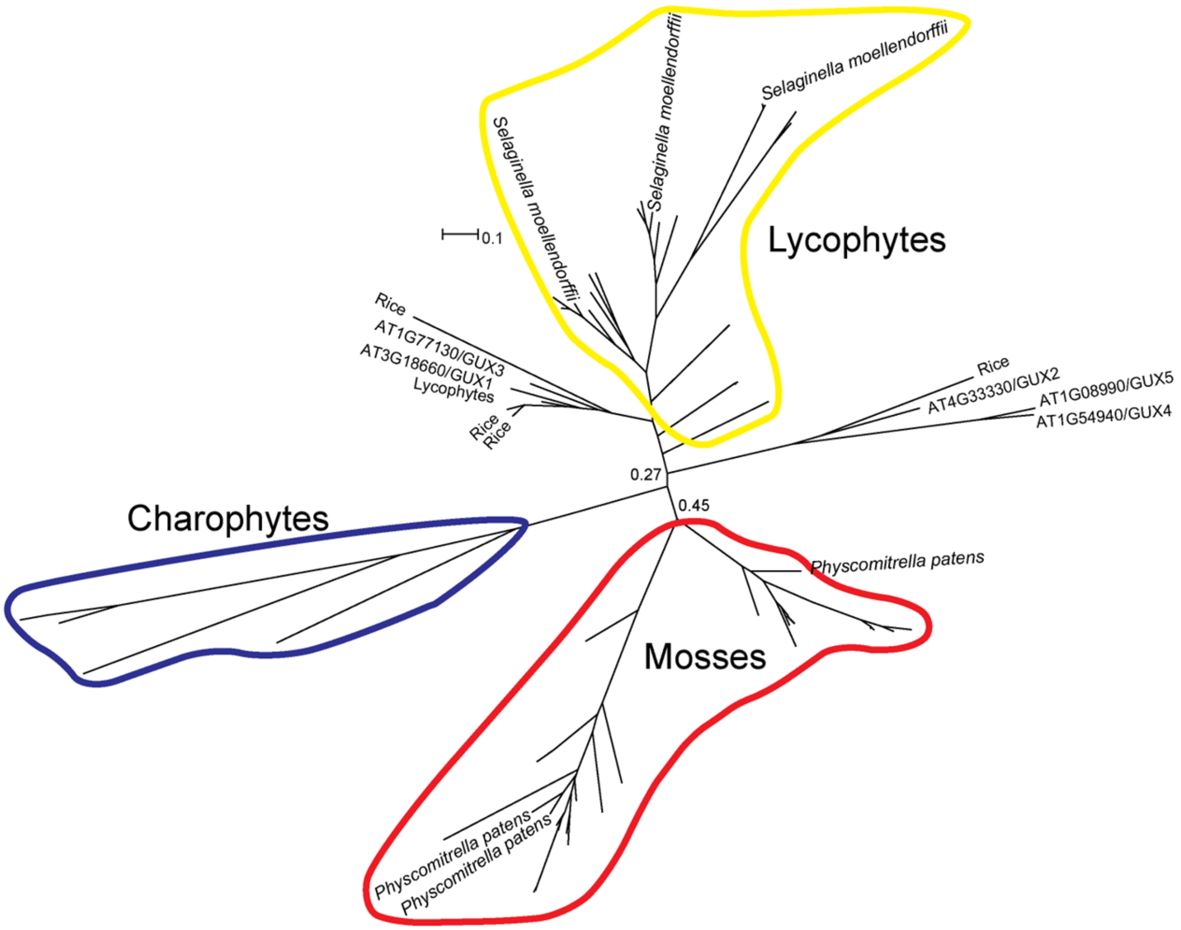

Fig. S6. Phylogenetic tree of GUX1-5 orthologs. Sequences included from charophytes, *Klebsormidium flaccidum*, *Physcomitrella patens*, *Selaginella moellendorffii*, rice and Arabidopsis. Encircling indicate sequences from Charophytes (dark blue), mosses (red) and lycophytes (yellow). Branches with no name correspond to charophyte, moss and lycophyte sequences from 1kp dataset and are probably not full length. All branches with names are sequences from complete genome projects. Branch support was evaluated with Approximate Likelihood-Ratio Test and is given as a value between 0 and 1. Supporting Information Notes S1

holds tree files where all sequences used are labeled.

Fig. S7 Phylogenetic tree of ESK1 orthologs.

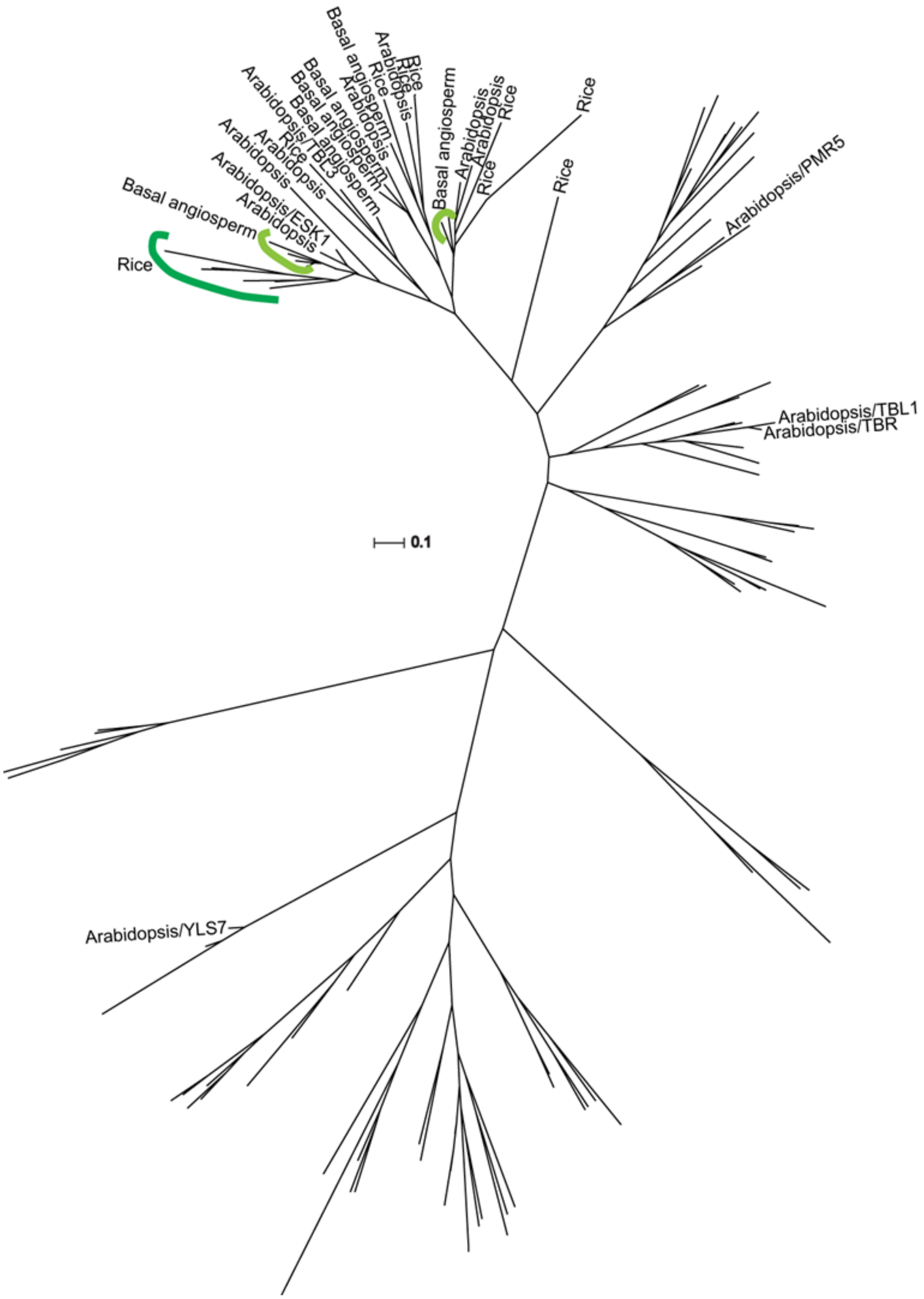

Fig. S7. Phylogenetic tree of ESK1 orthologs. Sequences included from basal angiosperms, rice, Arabidopsis and from the 1kp dataset. Encircling indicates sequences from rice (dark green) and basal angiosperms (light green). Branches in the TBL3 clade named basal angiosperm

correspond to basal angiosperm sequences from 1kp dataset and are probably not full length. Branches with no name are either rice or Arabidopsis full length sequences. Branch support was evaluated with Approximate Likelihood-Ratio Test and is given as a value between 0 and 1. Supporting Information Notes S1 holds tree files where all sequences used are labeled.

Fig. S8 Phylogenetic tree of GT61 orthologs.

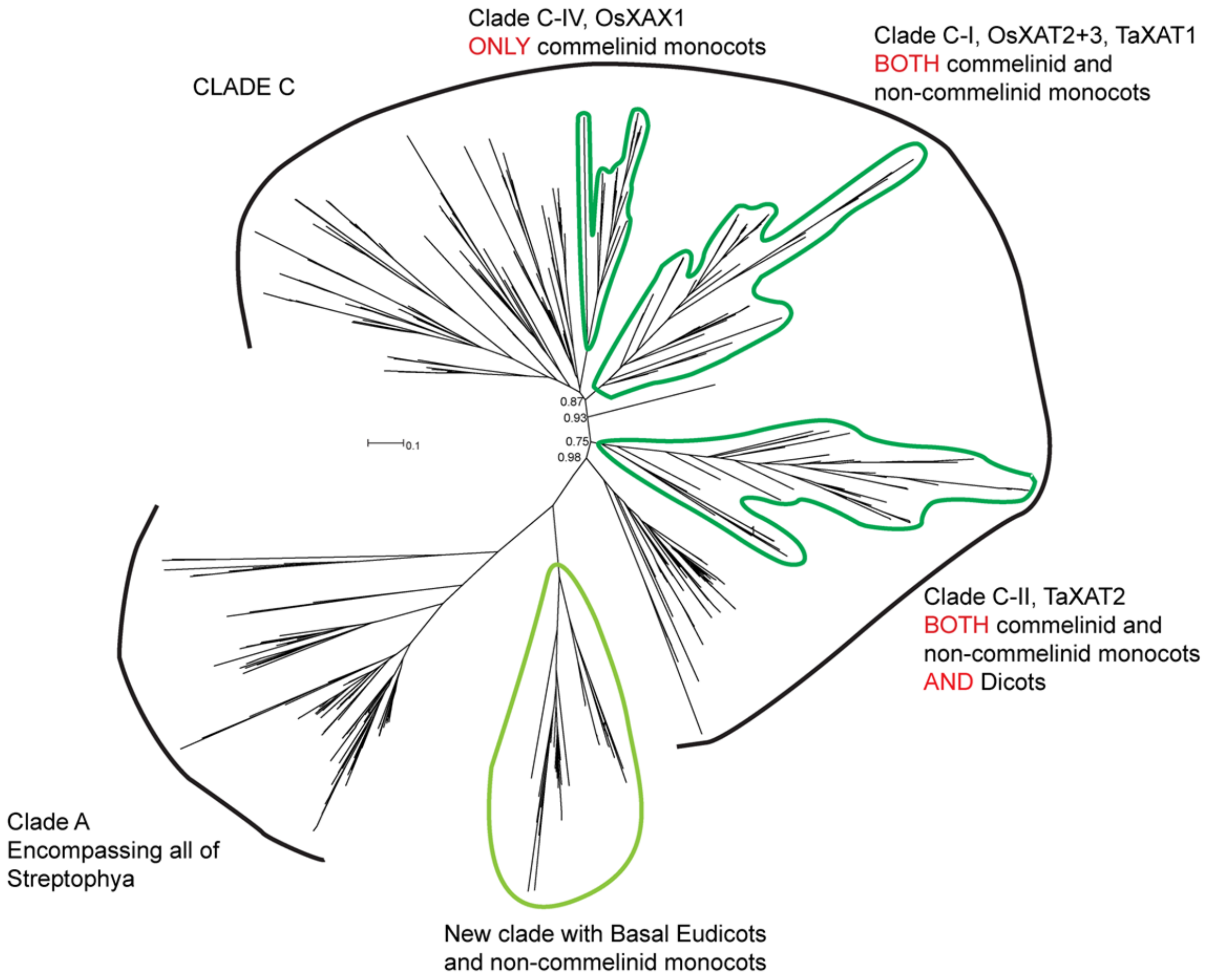

Fig. S8. Phylogenetic tree of GT61 orthologs. Sequences included from *Physcomitrella patens*, *Selaginella moellendorffii*, rice, Arabidopsis and angiosperms from the 1kp dataset. Clade B, encompassing AtXYLT, which is involved in N-glycan biosynthesis, is not included in the tree. Three subclades in the Clade C are individually encircled (green) and each hold at least one member of a proven xylan arabinosyltransferases (XA) from either rice or wheat. As indicated, these three clades contain commelinid monocots only (Clade C-IV), non-commelinid and

commelinid monocots (Clade C-I) and non-commelinid and commelinid monocots as well as dicots (Clade C-II). A new clade with basal eudicots and non-commelinid monocots were also identified (light green encircling). Branch support was evaluated with Approximate Likelihood-Ratio Test and is given as a value between 0 and 1. Supporting Information Notes S1 holds tree files where all sequences used are labeled.

Fig. S9 Phylogenetic tree of OsAT10 orthologs.

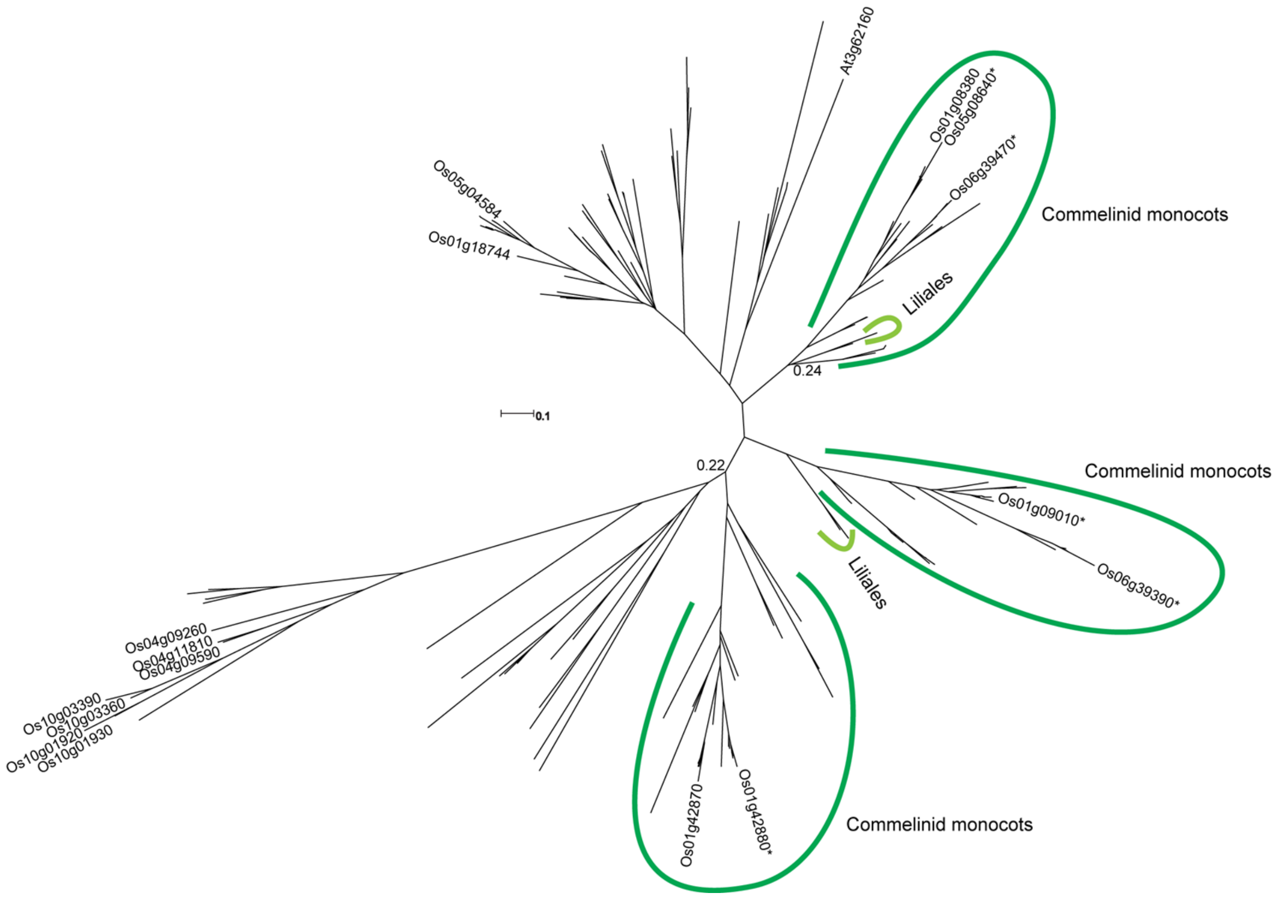

Fig. S9. Phylogenetic tree of OsAT10 orthologs. Sequences included from rice, Arabidopsis and monocots from the 1kp dataset. Rice OsAT10 is a putative cell wall related acyltransferase (BAHD) involved in ferulic or coumaric acid substitution of xylan. Sequences marked with asterisk are putative xylan ferulic or coumaric acid transferases. Encircling indicates sequences from a group of several species of commelinid monocots (dark green) and of lilioid monocots (light green). Branches with no name correspond to monocot sequences from 1kp dataset and are probably not full length. All branches with names are sequences from complete genome projects. Branch support was evaluated with Approximate Likelihood-Ratio Test and is given as a value between 0 and 1. Supporting Information Notes S1 holds tree files where all sequences

used are labeled.

Fig. S10 Enzymatic specificity of KfXYS1.

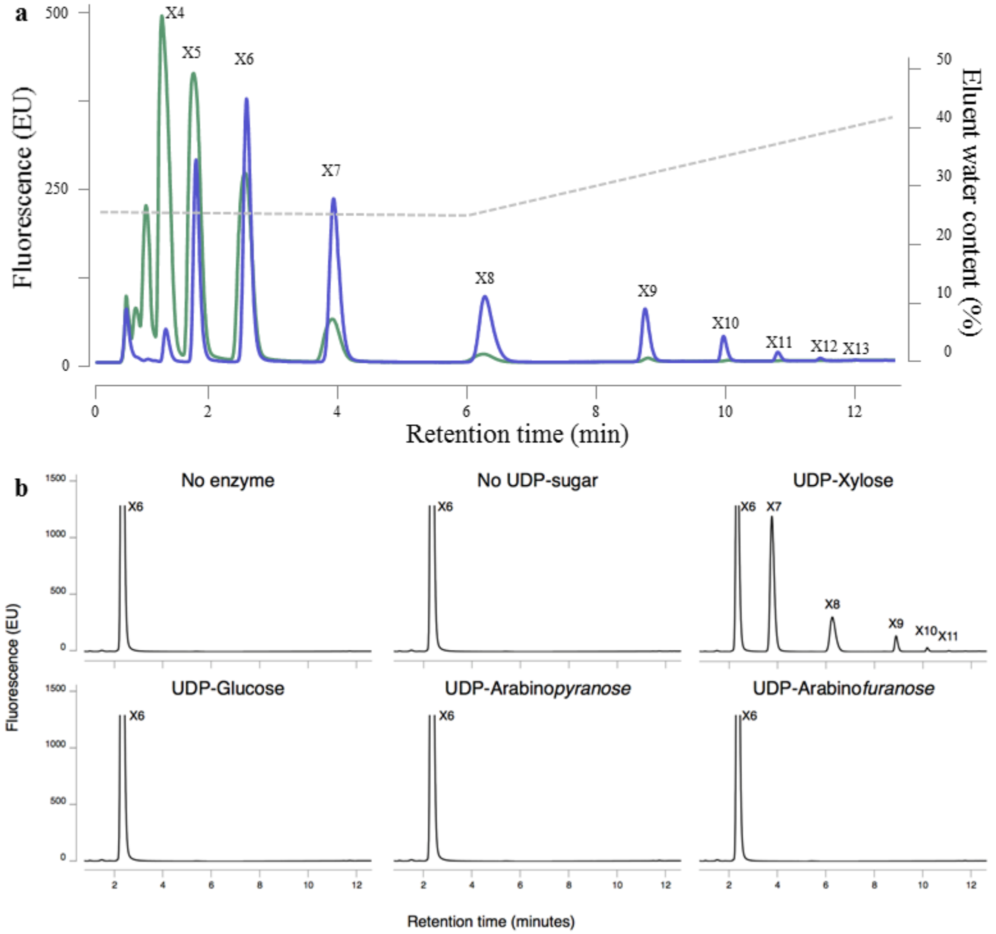

Fig. S10 Enzymatic specificity of KfXYS1. (a) KfXYS1 enzymatic product analysis by use of 1,4-β-xylanase. KfXYS1 protein was heterologously expressed in Saccharomyces cerevisiae. Reaction mixtures with UDP-xylose, fluorescently labeled xylotetraose (X4) and KfXYS1 preparation before (blue) and after xylanase treatment (green) were analyzed by HPLC. The shift in distribution towards X4-X6 shows that the xylanase is degrading areas of the oligos formed by KfXYS1 (from X4 and UDP-xylose) and so proves that the linkages formed are 1,4-β-linkages. Gray dashed line, eluent water content (right y-axis). X4-X13, progressively longer labeled xylan oligosaccharides.

(b) Donor substrate specificity of KfXYS1 protein heterologously expressed in *S. cerevisiae*. Reactions were performed with mixtures of the indicated UDP-sugars in combination with labeled xylohexaose (X6) and recombinant KfIRX10 protein preparation. Reactions were analyzed by HPLC.

Fig. S11 Characterization of reaction mixtures with or without KfXYS1 by 1D 1H NMR.

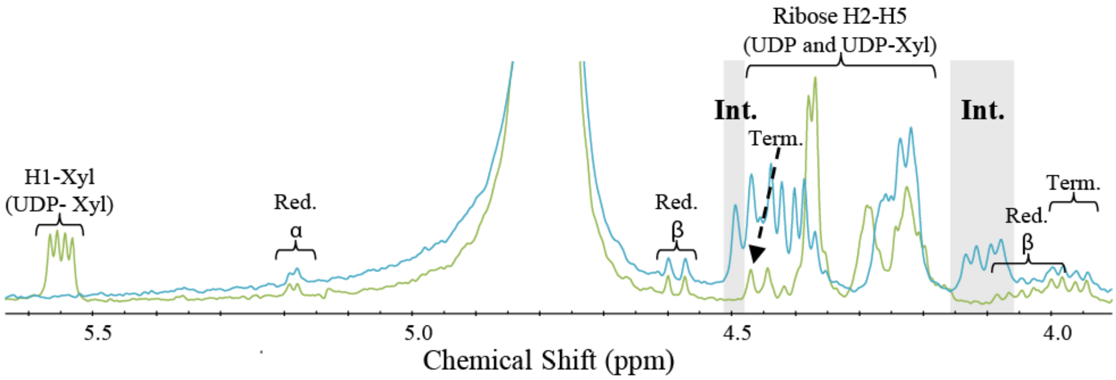

Fig. S11. Characterization of reaction mixtures with or without KfXYS1 by 1D 1H NMR.

The control spectrum of reaction mixture containing xylobiose and UDP-xylose only (green) contained signals of terminal (Term.) and reducing (Red.) xylose. KfXYS1 reaction spectrum (blue) after 16 h incubation containing diagnostic signals of internal β-1,4-xylose linkages (Int.; grey shade) that are absent in the control. Signals corresponding to H1 of the xylose of UDP-xylose in the control spectrum, are absent in the spectrum of the KfXYS1 reaction and only signals of UDP are present.

Table S1 Positive/negative list used for identification of xylan synthesis related sequences

ID	Function	AGI	Family	Subfamily	Name	Control1	Control2	Control3	Control4	Control5
At1g27440	Xylan beta-1,4-XylT	At1g27440	GT47	D	IRX10	At3g57630	At1g21480	At5g33290	At2g35100	At5g62220
At5g61840	Xylan beta-1,4-XylT	At5g61840	GT47	D	IRX10L	At3g57630	At1g21480	At5g33290	At2g35100	At5g62220
At2g28110	Xylan backbone related	At2g28110	GT47	D	IRX7	At3g57630	At1g21480	At5g33290	At2g35100	At5g62220
At5g22940	Xylan backbone related	At5g22940	GT47	D	IRX7L	At3g57630	At1g21480	At5g33290	At2g35100	At5g62220
At2g37090	Xylan backbone related	At2g37090	GT43	GT43A	IRX9	AAH71961	-	-	-	-
At1g27600	Xylan backbone related	At1g27600	GT43	GT43A	IRX9L	AAH71961	-	-	-	-
At4g36890	Xylan backbone related	At4g36890	GT43	GT43B	IRX14	AAH71961	-	-	-	-
At5g67230	Xylan backbone related	At5g67230	GT43	GT43B	IRX14L	AAH71961	-	-	-	-
At5g54690	Xylan backbone related	At5g54690	GT8	GAUT	IRX8	At3g61130	At2g20810	-	-	-
At1g19300	Xylan backbone related	At1g19300	GT8	GATL	PARVUS	At3g61130	At2g20810	-	-	-
At3g18660	Xylan alpha-1,2-GlcAT	At3g18660	GT8	GUX	GUX1	At5g18480	-	-	-	-
At4g33330	Xylan alpha-1,2-GlcAT	At4g33330	GT8	GUX	GUX2	At5g18480	-	-	-	-
At1g77130	Xylan alpha-1,2-GlcAT	At1g77130	GT8	GUX	GUX3	At5g18480	-	-	-	-
At1g54940	Xylan alpha-1,2-GlcAT	At1g54940	GT8	GUX	GUX4	At5g18480	-	-	-	-
At1g08990	Xylan alpha-1,2-GlcAT	At1g08990	GT8	GUX	GUX5	At5g18480	-	-	-	-
At1g33800	Glucoronoxylan 4-O-MethylT	At1g33800	DUF579	DUF579	GXMT1	-	-	-	-	-
At3g50220	Xylan backbone related	At3g50220	DUF579	DUF579	IRX15	-	-	-	-	-
At5g67210	Xylan backbone related	At5g67210	DUF579	DUF579	IRX15L	-	-	-	-	-
Os02g22480	Xylan alpha-1,3-AraT	Os02g22480	GT61	GT61CW	Os XAT1	At5g55500	-	-	-	-
Os03g37010	Xylan alpha-1,3-AraT	Os03g37010	GT61	GT61CW	Os XAT2	At5g55500	-	-	-	-
FR873610	Xylan alpha-1,3-AraT	FR873610	GT61	GT61CW	Ta XAT1	At5g55500	-	-	-	-
FR846232	Xylan alpha-1,3-AraT	FR846232	GT61	GT61CW	Ta XAT2	At5g55500	-	-	-	-
Os02g22380	Xylan arabinosyl beta-1,2-XylT	Os02g22380	GT61	GT61CW	Os XAX1	At5g55500	-	-	-	-
At3g18170	Putative xylan AraT	At3g18170	GT61	GT61CW	Closest At to XAT1	At5g55500	-	-	-	-
At3g18170	Putative xylan AraT	At3g18170	GT61	GT61CW	Closest At to XAT1	At5g55500	-	-	-	-
At3g55990	Xylan acetyltransferase	At3g55990	TBR/TBL	TBR/TBL	TBL29	At5g58600	-	-	-	-
Os06g39390	Xylan coumaroyl transferase	Os06g39390	BAHD	BAHD	Os AT10	At3g48720	-	-	-	-
At3g62160	-	At3g62160	BAHD	BAHD	Closest At to OsAT10	At3g48720	-	-	-	-

Table S2 Protein sequences of *Klebsormidium flaccidum* xylan gene orthologs.

**Supplementary Table 1.** Protein sequences of *Klebsormidium flaccidum* xylan gene orthologs	
IRX10/IRX7 orthologs	
kfl00398_0130 / KfIRX10	MPNKLFTGFAALFCFCFFLLGSIIQRSSLFVLDVENKATVQAAERELTERTWDVQEGTTGVVARRALLDRLMLEEEVPEDDEEDKVPSVLRESPEGRLKVFVYDLPSKYNTKLLEKDSRCLNHMFA<br>AEIFVHRNLLNSSIRTMDPSEADWFYTPVYTTCDLTPNGLPLPFKAPQLMRSAIRWVAEHYPYWNRSEGADHFFTVPHDFGACFHYQEEEAIKRRILPVLQRATLVQTFGQNNHVCLKEGSIVV<br>PPYAPPQKMRSRFIPPDTPRSIFVYFRGMFYDISNDPEGGYYARGARAAIWENFKDNPLFDLSSDHPSTYYEDMQRAVFCLCPLGWAPWSPRIVESVIFGCIPVIIGDDIVLPFSDAIRWDEISVY<br>VAEKDVQNLDTILTAIKPEEIMAMQRKMWKPEVRTAMLFPQPAEPGDSFHQILNGLARKLPHKPEVFVKPGEHPIDWTAGPQQDQDEWRGDDEN
kfl00254_0250	MPPSDATLGRAGSMSNASGRSVSPATNRRCVCGYCAPSSFCTCASSPAPSTGESLGGLQRSSSQMSLTSAFSSKQLRKQASSTRLDQGAFFYFRQRLAFSCTGLMLVMLCVALASRHLQHASV<br>DGLERVPPFRDSRRMMMSADDGISVSTGTTAVIQNGLESTGLALPKEVIPNSPLGGPLLEAPARKYNAIASQMKIYIYDLPSYFNADLVTRDPRILTHMFAAEVLIHYSLMNSSVRTMNPDEADL<br>FFIPVYSTGDLNDQGRALPQMAGMMIKQVMHHVVHTWPFFNRTHGSDHVVVVPHDFGACFTFREEEAVTRKVPAVLRKLLMLQTFGQENHTCSRSGRSIIIPPYVNVNWVKSVPGLPEGP<br>GQPPRDKFAYFRGLVHDFGNDPWGNKYSKGVRYQIWKHHKDNPLFDVAEMVPFAYYEDMRRSVFCLCPLGWAPWSPRIVESLQQGCIPVIISDNIILPFREFVDWPAITVHVPEKDVPRLDEI<br>LSAIPVEEVAAMQKRIWSPAVRRAVLFPQPAEEGDAFHYVLRELARKLEQRREAQREKDRKSKSKKKSPTNALRGESQTAALHALR
kfl00144_0240	MGCGRRWIAVNVVGVCLLLLVFGGVVGPDLTQSGLGPGDSKSGRVSGVGHGRRARALLQSRRALQQTDRTFARERRRWDAEALQNVGVPVILGRGVSVEDRVLHFRRRGISDFFEAEPEWP<br>ESQESEEPEDEVGAESSMEGDTDQVLDSKSVGEAGSGKTGHKGLGKGGPGSVPELIIRDSDSEVRTAETFAPVQTLFVNVTANSTGGAANSSNLEKKGPLLGQGLPLNITGGVATNKSSRLNQS<br>EFSSRDPDFDAHPHILQNVTEAERVEQTTAESEPLTKGAPGEVRDIEQEQGLELEEIETTAPTQPPEEAEMQNPFADPETAPMLVLPPQRSIKVYIYDLPPHLNRDFVTRDPRVRQTMFATEVLIH<br>ENFEQSAVRTLDPDEADFYFIPVYTAAVLEDSGRTIPDSSHGTMLEAVMYVAERWPYFNRMRGLDHVVVAPHDYGACFHYRDELASQTKFRMVLRYLTVLQTFGVHNHVCYRPGRSVVIPPYI<br>DVRKLRQLAFAPERDAHEKSVLAFFRGLYIDAGNDATGKVYSRGVRLAIGQTFANHPDFDISDAPSLTYLQDMMRATFCLCPLGWAPWSPRIFESVQIGCIPVIIADDIILPFENYFNWERIAVRV<br>KEADVPRLEEILKGIPPEKVAAMQEAMWDPQVRQALIFPEPAEPGDAFEMILNELEAKVERIRHKRAEYFRKHGQ
kfl00113_0290	MHGVKLPRLTTTSSGNHSKQPHRRGPSYSKRRAPLRRWIRRLGGCTVCCASVILFLLMSAVVFLAVKSWPGAAVSSAFDLGTDESPANVWRRGGERGGEHDAEEWGGGEIAKGLDLGAYTA<br>MVGGDVAPPTSIADLGLPVPLRIYVYELPPSFNVDFLRNPRCASHLFAAEVAIHRALLQSPVRTLQPAEADFFFVPVYTACNFNTENGFPNLSGAPEQLRTAVELLAAEYQYWNRTNGRDHVFV<br>ATHDFGACFHAMEKAAVQAGVPQFLRNSIILQTFGQTNNHPCQDVDHIQIPPYVPSPLVLANWQPPESQNRTIFAYMSGKIEVHPKESVSGDVYGRAVRTGIWKRFHRDKRFLIRRKRPEAYY<br>EEMLHARFCLCPRGWAPWSPRIVESVLFGCIPVIIADHIKLPFDDLLNWPAMGLILQENEVPNLATILESLSEDRVASMQRELWRAEVRRTLLFLEPSVRGDATWQILDALAKKKMERDKLQ
IRX8 ortholog	
kfl00095_0020 / KfIRX8	MKLKLSPSMRRITITAQEDFHPMGLKSAPACCSYSCVMVSLLGLALILPMLFITTAIVKLDSIDNCIDGECYTRGRKMSPTDRADELVTGLLDVPVSVGRQLNEERTGVGGDAVVGEREETIDDLI<br>AQVSELNDDSASVILKLRALVEVHEGKARMAKLQRALYRHFASKGVPKGLHCLSLRLTADYSTNPEANRPLAGPEEISRLTDNSLRHFVLLSDNVLATSVVVNSTVTNAKEPEKVVFHVVTDETTY<br>SAMHAWFALFPPEPATIEVRGVHEMQWLTAAKAPVLDLMENSLALQKLNYGDRILEEAANDPPQVLAAKLQARSPKYISIMNHLRMYLPEVFPELDRIIFLDDDVVVQKDLTGLWEMDMRG<br>KVNAGVETCRGADRFALSKQFRSYFNFSNPLIAEHFAPETCAWAFGLNVFDLKRWRESNITETYHHWQKQNMMLNMTLWRLGTLPPALIAFYGHTLPLDHSWHMLGLGYQPDSSIEVIKSSA<br>VIHYNGPAKPWLDMAFTDFRSYWTRWVDYGNEFVHQCNIFQMRMMGKRKAPSSSSTPPGQKAKGPRQDSGAADGSLPERRSFVHGSAFLIVCFACFLLGFRLSWEGKAAFLGAYRIGPES<br>GKLVLRDTGTSLDYADSESGGGAAQGGLDLRPGAWESGQGDKYGAGVADGEWERIETRILASAGGVVNASESNRSRRLLPRPPPSPPEHPWVGRHNISVRPWPHPDAGETLLAHALIAAVQ<br>REQQNQFGVPLDRRKRMIVVTPTYRRAFQQIHLSHLTDTLRLAPEPLSWLVVEAGGPTEETQAILSKTGLADIHLLPVPGVTMPDVRAPERPIVEDYLRDVALGYIQKQAMEGVVVFADDSATL<br>RLALFQELQKVRWFAAFSVGMLVQGGGSQVDLPVQGPVCGPEGDVRGWHAADHELLRLKKPAVVPKRALLGAAEKRLPGGEGAQRRLLALEVPERTEGFGAGSDSISQLGAGTVVRQLQQ<br>VEQSEYFEPELFIPKLEWVGFAFNARLLWAPAERPAWLREWKPSTTLGKVDAVVGDYEGDEDPTDLLARMVEDESQVETLGRCGKDVLAWWLKVEAAEEGQYPAGWRLAENLPVVAEPQE<br>WSEEQWAEYEARLVEEKKKKKKKSKKKKKAPEATKVEEDAAARQAAEQAAADEARRVEEEKQRELEAARQAEEERQTRLAEAQKAEVEDNLDSVQKAKAEVRTDPEKEGEKAESKLESSALA<br>EKEALTNNAKEVLNKLVEGADGGVADDTQAREKQTS
IRX9 ortholog	
>kfl00579_0060 / KfIRX9	MPPGGGKPMAKHAPIIGSLLNNRRPLLLGFCFLLGFLLGVFDTRRFEASFPDAVSHSFPGQALEDEQRGLVGAGASRILVQQQLPAQPRLEAPVQLLIVTPTYNRPFQAMYLTRLAHTLRLVRPP<br>VLWIVVETGWQSEESVALLMRTGLMYRHLVVQQFSPQHKDRGVHQRNHALDHIQQHALQGIVYFADDDNNYSVELFEEIRKVKRFGVLPVAMLVEGAEKTTIEGPVCAGGRVIGWHTSEES<br>AAQRRFHSDLCGFAFNSSLMWEPGGEGRRRNAPVRFRDNIKEGFQETTFIEQLVGSEAEMEGLADDCREVLVWHLRAEAARDSVYPTSWMLRASMNITTPARTPESIREP
IRX14 ortholog	
kfl00262_0200 / KfIRX14	MMGKRKAPSSSSTPPGQKAKGPRQDSGAADGSLPERRSFVHGSAFLIVCFACFLLGFRLSWEGKAAFLGAYRIGPESGKLVLRDTGTSLDYADSESGGGAAQGGLDLRPGAWESGQGDKYGA<br>GVADGEWERIETRILASAGGVVNASESNRSRRLLPRPPPSPPEHPWVGRHNISVRPWPHPDAGETLLAHALIAAVQREQQNQFGVPLDRRKRMIVVTPTYRRAFQQIHLSHLTDTLRLAPEPLS<br>WLVVEAGGPTEETQAILSKTGLADIHLLPVPGVTMPDVRAPERPIVEDYLRDVALGYIQKQAMEGVVVFADDSATLRLALFQELQKVRWFAAFSVGMLVQGGGSQVDLPVQGPVCGPEGDV<br>RGWHAADHELLRLKKPAVVPKRALLGAAEKRLPGGEGAQRRLLALEVPERTEGFGAGSDSISQLGAGTVVRQLQQVEQSEYFEPELFIPKLEWVGFAFNARLLWAPAERPAWLREWKPSTTLG<br>KVDAVVGDYEGDEDPTDLLARMVEDESQVETLGRCGKDVLAWWLKVEAAEEGQYPAGWRLAENLPVVAEPQEWSEEQWAEYEARLVEEKKKKKKKSKKKKKAPEATKVEEDAAARQAA<br>EQAAADEARRVEEEKQRELEAARQAEEERQTRLAEAQKAEVEDNLDSVQKAKAEVRTDPEKEGEKAESKLESSALAEKEALTNNAKEVLNKLVEGADGGVADDTQAREKQTS

Table S3 Protein BLAST analysis of Klebsormidium flaccidum xylan gene orthologs

Supplementary Table S2. Protein BLAST analysis of *Klebsormidium flaccidum* xylan gene orthologs

Protein name	Max score	Total score	Query cover	E value	Ident
**IRX10L**	**860**	**860**	**1**	**0**	**1**
Potri012G109600	778	778	0.95	0	0.93
PoIRX10_5	764	764	1	0	0.88
IRX10	750	750	1	0	0.86
Potri003G162000	741	741	0.93	0	0.9
Potri001G068100	738	738	0.99	0	0.85
Os01g70200	732	732	0.96	0	0.86
PoIRX10_6	730	730	0.95	0	0.86
Bd2g59410	720	720	0.93	0	0.87
Potri012G109200	719	719	0.95	0	0.88
Os02g32110	719	719	0.93	0	0.88
Os01g70190	716	716	0.94	0	0.85
Sm442111	711	711	0.93	0	0.87
Bd2g59400	711	711	0.93	0	0.85
Os04g32670	711	711	0.93	0	0.86
Bd5g08400	710	710	0.93	0	0.86
PoIRX10_3	708	708	0.98	0	0.81
Bd3g44420	703	703	0.93	0	0.85
Pp1s7_455V6	674	674	0.99	0	0.77
Bd2g59380	667	667	0.92	0	0.8
Os01g70180	650	650	0.94	0	0.77
Os10g10080	644	644	0.98	0	0.74
**kfl00398_0130 / KfIRX10**	**620**	**620**	**0.9**	**0**	**0.75**
PoIRX10_1	593	593	0.97	0	0.69
PoIRX10_2	573	573	0.88	0	0.73
PoIRX10_4	566	566	0.91	0	0.71
**kfl00254_0250**	**379**	**379**	**0.83**	**4E-130**	**0.5**
**kfl00144_0240**	**345**	**345**	**0.84**	**2E-115**	**0.47**
**kfl00113_0290**	**314**	**314**	**0.81**	**4E-106**	**0.46**
FRA8	299	318	0.87	1E-100	0.42
XGD1	103	103	0.62	4E-27	0.3
**AtIRX8**	**1112**	**1112**	**1**	**0**	**1**
Potri.001G416800	901	901	1	0	0.82
Potri.011G132600	871	871	0.99	0	0.81
Potri.004G124500	634	634	1	0	0.55
Potri.017G090800	622	622	1	0	0.54
Bradi4g03670	596	596	0.99	0	0.54
LOC_Os12g38930	583	583	0.95	0	0.55
Potri.004G124500	574	574	0.87	0	0.57
Bradi1g70290	571	571	0.99	0	0.51
Bradi1g70290	571	571	0.99	0	0.51
LOC_Os03g11330	566	566	0.99	0	0.5
**kfl00095_0020_IRX8**	**478**	**478**	**0.99**	**2E-159**	**0.43**
**AtIRX9-L**	**823**	**823**	**1**	**0**	**1**
**kfl00579_0060_IRX9**	**288**	**288**	**0.74**	**8E-99**	**0.49**
Potri.016G086400	244	244	0.79	9E-82	0.42
Potri.006G131000	242	242	0.79	1E-80	0.41
AtIRX9	218	218	0.77	1E-71	0.39
Bradi2g37970	172	172	0.79	2E-53	0.34
LOC_Os05g03174	169	169	0.81	2E-52	0.33
LOC_Os04g58040	50.4	68.9	0.26	1E-10	0.39
**AtIRX14**	**1082**	**1082**	**1**	**0**	**1**
AtIRX14-L	652	652	0.96	0	0.65
Potri.007G047500	642	642	0.88	0	0.7
Potri.005G141500	640	640	1	0	0.65
Potri.007G047500	589	589	0.83	0	0.69
Bradi1g33320	480	480	0.8	2E-169	0.54
LOC_Os06g47340	475	475	0.8	2E-167	0.55
LOC_Os04g55670	246	246	0.84	2E-79	0.36
**kfl00262_0200_IRX14**	**206**	**350**	**0.8**	**4E-62**	**0.35**

Pairwise protein sequence comparison was performed using BLAST (Altschul et al., 1997). For each sub section of the table the proteins are compared pairwise with the top entry maked in bold. *K. flaccidum* proteins are indicated in red.

Notes S1 Newick tree files and sequences used for Fig. 2 and Fig. S2-S9.
